# Active Sampling State Dynamically Enhances Olfactory Bulb Odor Representation

**DOI:** 10.1016/j.neuron.2018.05.016

**Published:** 2018-06-27

**Authors:** Rebecca Jordan, Izumi Fukunaga, Mihaly Kollo, Andreas T. Schaefer

**Affiliations:** 1 Neurophysiology of Behaviour Laboratory, Francis Crick Institute, London NW1 5AT, UK; 2 Department of Neuroscience, Physiology & Pharmacology, University College London, London WC1E 6BT, UK

**Keywords:** olfaction, learning, context, olfactory bulb, active sampling, sniffing, behavior

## Abstract

The olfactory bulb (OB) is the first site of synaptic odor information processing, yet a wealth of contextual and learned information has been described in its activity. To investigate the mechanistic basis of contextual modulation, we use whole-cell recordings to measure odor responses across rapid learning episodes in identified mitral/tufted cells (MTCs). Across these learning episodes, diverse response changes occur already during the first sniff cycle. Motivated mice develop active sniffing strategies across learning that robustly correspond to the odor response changes, resulting in enhanced odor representation. Evoking fast sniffing in different behavioral states demonstrates that response changes during active sampling exceed those predicted from feedforward input alone. Finally, response changes are highly correlated in tufted cells, but not mitral cells, indicating there are cell-type-specific effects on odor representation during active sampling. Altogether, we show that active sampling is strongly associated with enhanced OB responsiveness on rapid timescales.

## Highlights

• During rapid olfactory learning, responses of mitral and tufted cells change overtly
• These changes reliably track the development of active sniffing
• This enhances odor detectability and discriminability in a cell-type-specific way
• Active sniffing impacts odor responses through both bottom-up and top-down mechanisms

## Teaser

Using whole-cell recordings in the olfactory bulb of mice across a range of behavioral states, Jordan et al. show that odor responses are dynamically enhanced during learning and task engagement, tightly mirroring alterations in active sniffing.

## Introduction

The ability to respond to sensory stimuli according to learning and context is vital for orchestrating appropriate behavior. Our view of sensory processing has shifted away from the simplicity of passive feedforward models driven by sensory stimuli, to one that additionally incorporates contextual information provided by top-down circuits into the ongoing processing (Engel et al., 2001). This has been driven in part by observations that activity in primary sensory cortex is widely modulated by contextual information: locomotion, attention, and experience all modulate visual cortex activity (Fiser et al., 2016, Ito and Gilbert, 1999, Niell and Stryker, 2010), while whisking behavior and social context modulate barrel cortex activity (Lenschow and Brecht, 2015).

The olfactory bulb (OB) is the first site to synaptically process olfactory information, yet already modulation by multiple contexts has been described in recordings of suprathreshold activity (unit recordings and imaging). These include modulation of odor responses by hunger state (Pager et al., 1972), task engagement (Fuentes et al., 2008), reward association (Doucette and Restrepo, 2008), conditioned aversion (Kass et al., 2013), and even non-olfactory events (Kay and Laurent, 1999, Rinberg et al., 2006a). Recently, several studies have described changes in mitral and tufted cell (MTC) odor responses during olfactory learning (Chu et al., 2016, Doucette and Restrepo, 2008, Yamada et al., 2017). Despite the prominence of such studies, the mechanistic basis underlying contextual modulation of the circuit is still unclear. In particular, rarely have contextual modulations been interpreted in the framework of active sniffing behavior, which is known to be controlled in a highly context-dependent manner (Wachowiak, 2011). Rodents often show development of sniffing strategies alongside the learning of olfactory discrimination tasks (Kepecs et al., 2007, Wesson et al., 2008, Wesson et al., 2009, Youngentob et al., 1987), and the impact of this on odor representation is currently not well understood. Additionally, while it is possible to chronically record across long timescales using unit recordings and imaging, they have limited access to subthreshold activity, while the former may under-sample from cells with low firing rate (Kollo et al., 2014, Margrie et al., 2002) and have difficulties with cell-type identification.

We thus wanted to investigate to what extent active sampling strategies account for context-dependent changes in MTC odor responses. To this end, we recorded from identified MTCs using blind whole-cell recordings *in vivo* across a range of behavioral states. We optimized behavioral training protocols to facilitate rapid olfactory discrimination learning, which allowed us to make whole-cell recordings over the full learning epoch and simultaneously measure sniffing behavior. Altogether, we show that changes in active sniffing behavior are strong predictors of odor response change. These response changes exceed those predicted by feedforward input alone, occur in a cell-type-specific way and overall enhance the representation of odors within the OB.

## Results

### Whole-Cell Recordings during Go/No-Go Discrimination Learning

**Figure 1:**
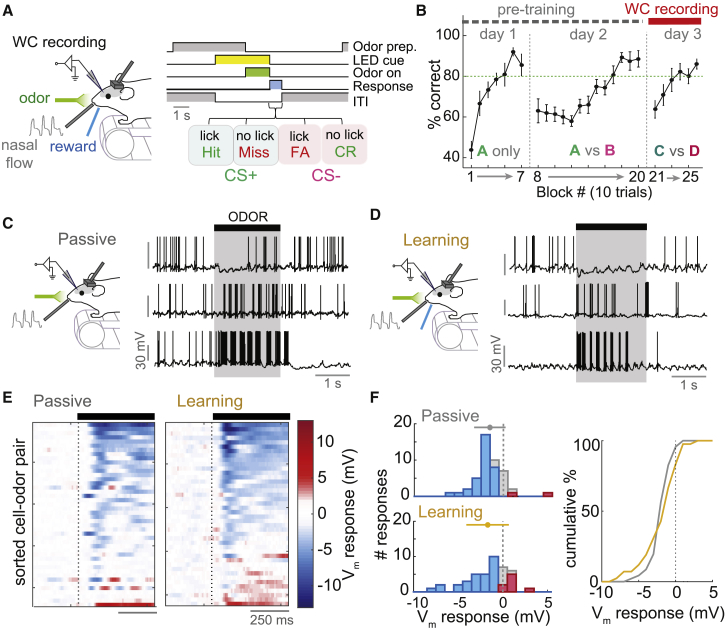
Paradigm for Behavioral Task and Whole-Cell Recording across Learning (A) Diagram of the whole-cell (WC) recording setup (left) and schematic of go/no-go task sequence (right; described in text). (B) Average learning curves for 21 mice for pre-training sessions (day 1, operant conditioning to the CS^+^; day 2, discrimination learning for the first odor pair) and discrimination learning with a novel odor stimulus pair on day 3. Whole-cell recordings were made across this learning epoch. Green dashed line indicates criterion of 80% correct. Error bars show SD. (C) Example V_m_ traces during odor stimulus for cells recorded in passive mice, aligned to first inhalation onset. (D) As for (C), but for cells recorded in learning mice. (E) Heatmap of V_m_ responses averaged across all trials for each cell-odor pair, sorted by mean V_m_ response, for both passive (n = 46) and learning (n = 42) datasets. Black bar indicates odor stimulus, aligned to first inhalation onset. (F) Left: histograms of average 500 ms V_m_ responses for passively exposed (top) and learning mice (bottom). Red bars indicate significant excitation; blue bars indicate significant inhibition. Error bars above histogram show mean ± 1 SD. Right: cumulative histograms comparing average V_m_ responses for passive (gray) and learning (gold) cell-odor pairs. Passive, V_m_ response = −1.5 ± 1.8 mV; learning, V_m_ response = −1.7 ± 2.4 mV; p = 0.05, Bartlett test.

To observe changes in odor response across olfactory learning, we recorded from 21 MTCs in mice during learning of a simple olfactory go/no-go discrimination task (Figure 1A). In this task, two odor mixture stimuli (Figure S1) were randomly selected and presented in a pseudorandom sequence, each preceded by an LED cue. When the presented stimulus was the assigned CS^+^, mice were required to lick the reward port in the 1 s after odor offset to gain a reward of dilute sweetened condensed milk, and to avoid licking if the stimulus was the assigned CS^−^ to prevent a time addition to the inter-trial interval (ITI). Mice were pre-trained prior to recording on different odor pairs (Figure 1B; STAR Methods), until reaching criterion (>80% correct) for odor discrimination. This pre-training allowed mice to undergo much more rapid learning on the subsequent novel odor pair, reaching criterion within 20 min (Figure 1B). Whole-cell recordings took place across this rapid learning epoch. MTCs were distinguished from interneurons as previously described (Kollo et al., 2014), using independent component analysis of the spike after-hyperpolarization (AHP) waveform, and confirmed with morphological reconstruction of 11 cells (Figures S3A–S3C; STAR Methods).

To control for time-dependent effects unrelated to discrimination learning, we also recorded from 23 MTCs in a separate cohort of mice that were passively exposed to repeated presentations of the same odor stimuli. To first test whether the learning and passive states show any general difference in OB physiology, we applied a series of current steps and compared the basic properties of cells. Both the passive properties (input resistance, membrane time constant, and resting V_m_) and spontaneous activity of cells revealed few detectable differences (Figures S4A–S4F).

The odor mixtures used as stimuli were intended to activate a large portion of the dorsal OB (STAR Methods). These mixtures evoked diverse MTC responses in both passive (Figure 1C; 46 cell-odor pairs) and learning mice (Figure 1D; 42 cell-odor pairs). Note that all odor responses in the study are aligned on each trial to the first inhalation onset, and we have included both CS^+^ and CS^−^ cell-odor pairs in all analyses unless otherwise stated. Averaging V_m_ responses (membrane potentials calculated after spike-subtraction; Figures S3D and S3E) across all trials revealed that over 80% of cell-odor pairs responded to the odor mixtures (Figures 1E and 1F). Firing rate (FR) responses were also evoked with similarly high probability (Figures S4G and S4H).

### Diverse Odor Response Changes Occur in Learning Mice

**Figure 2:**
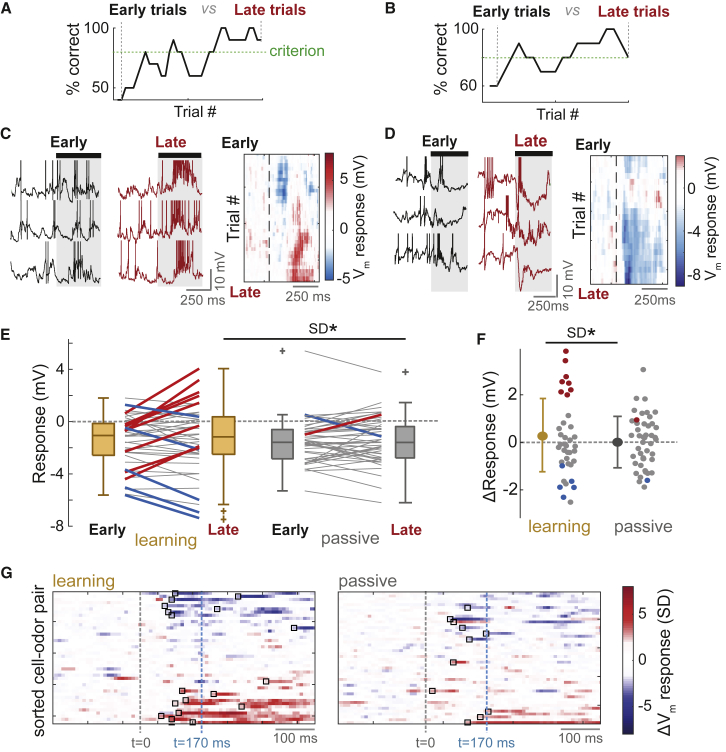
Diverse Odor Response Changes Occur in Learning Mice (A) Example learning curves for one mouse across the recording. Percentage correct is calculated in a moving average of five CS^+^ and five CS^−^ trials. Odor responses are compared between five early trials (unlearned) and five late trials (learned) to assess learning-related changes. (B) As for (A) but for a different mouse. (C) Left: example odor response V_m_ traces in early and late trials for a cell-odor pair undergoing an increase in excitation across learning (spikes have been clipped for display). Black bar and shaded area indicate odor stimulus (aligned to first inhalation onset). Right: heatmap showing five-trial moving average of V_m_ response. Note that this example corresponds to above learning curve in (A). (D) As for (C), but for a response undergoing an increase in inhibition (corresponds to B). (E) Plot between mean early and late V_m_ responses for learning mice (left; n = 42 cell-odor pairs) and passive mice (right; n = 46 cell-odor pairs) separately. Thick red and blue lines indicate significant positive and negative change, respectively (p < 0.01, unpaired t tests). Response variance did not differ in early trials (learning versus passive, p = 0.17, Brown-Forsythe test) but did during late trials (p = 0.03, Brown-Forsythe test). (F) Comparison of response changes (late-early) for learning and passive mice. Red and blue dots show significant positive and negative changes, respectively. Error bars show SD. (G) Response change heatmaps (late-early mean V_m_ response waveforms) normalized by baseline SD. Black boxes indicate onset of change (>2 or <(−2) SD for at least 50 ms). t = 0 indicates odor onset, aligned to the first inhalation onset. t = 170 ms is indicated as the minimal reaction time (Figure S2).

Recent imaging studies show that MTC responses can change over long timescales in both learning and passive mice (Chu et al., 2016, Yamada et al., 2017). Comparing V_m_ responses averaged across all trials revealed that response variance was larger across cells in learning mice relative to passive mice (p = 0.05, Bartlett test; Figure 1F). To assess whether this difference developed across rapid learning, we compared the V_m_ response of each cell-odor pair in five early trials (#2 to #6), in which the mouse is performing at chance levels, with the response in the five last trials, in which the mouse is performing at criterion or above (e.g., Figures 2A and 2B). Since median reaction times in the task were 500 ms (Figure S2A), we focused on the first 500 ms of the odor response for all analyses (unless otherwise stated).

Diverse changes in V_m_ response occurred over the course of learning, including increases in excitatory response (Figure 2C) and increases in inhibitory response (Figure 2D), which emerged gradually across trials (Figures S5C and S5D). Overall, in learning mice, 30% of cell-odor pairs underwent a significant response change across learning (p < 0.01, unpaired t test between 5 early and 5 late trials), with 19% showing a positive change and 11% showing a negative change (Figures 2E and S5A). The V_m_ response changes often gave rise to changes in FR: increases in excitatory V_m_ response were reflected by increases in excitatory FR response, though this was less clear for changes in inhibition (Figures S6A–S6E). MTCs recorded in passive mice, in which recording durations were matched (Figure S5E), showed significantly less response changes than learning mice (2/46 passive versus 13/42 learning cell-odor pairs, p = 0.001 Fisher’s exact test; Figures 2E and S5B). Overall, there was significantly higher variance in response changes for learning compared to passive datasets (learning ΔV_m_ SD = 1.5 mV; passive ΔV_m_ SD = 1.1 mV; p = 0.02, Bartlett test; Figure 2F). This dichotomy in response changes meant that in late trials, the learning dataset showed significantly more variance in responses than the passive dataset, while this was not the case in early trials (Figure 2E). Thus, response differences developed across the learning episode and were not due to general behavioral state differences, such as thirst state.

Response changes did not reflect the contingency of the odor: in fact, response changes for CS^+^ and CS^−^ stimuli were correlated (Figures S7A–S7E). The changes also occurred within a behaviorally relevant time window: mice could have reaction times as low as 170 ms (Figure S2), congruent with previous estimates (Abraham et al., 2004, Resulaj and Rinberg, 2015, Uchida and Mainen, 2003), and 67% of identifiable V_m_ response changes occurred prior to 170 ms (median ΔV_m_ onset = 120 ms, interquartile range [IQR] = 90–220 ms; Figures 2G and S5F; STAR Methods).

Thus, diverse response changes occur specifically across learning within a behaviorally relevant time window.

### Active Sampling Strategies Emerge across Task Learning

**Figure 3:**
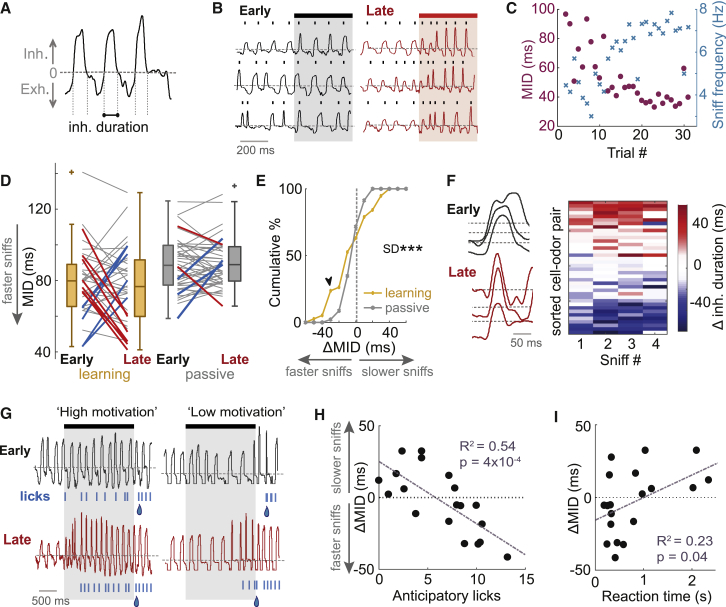
Active Sampling Strategies Emerge across Task Learning (A) Diagram to show extraction of inhalation duration from example nasal flow trace. (B) Example nasal flow traces from one mouse showing emergence of rapid sniffing between early and late trials. (C) MID for example in (B) calculated for each trial (first 500 ms of stimulus) in purple dots. Blue crosses show corresponding sniff frequency for each trial. (D) Plot showing how MID changes between early and late trials, for learning (n = 38) and passive (n = 42) mice. Thick red lines show significant reductions in MID (faster sniffing); thick blue lines show significant increases in MID (slower sniffing; p < 0.01, unpaired t tests). (E) Cumulative histograms of MID change (late-early) for learning and passive mice. Black arrowhead shows point of significant difference (STAR Methods). (F) Left: example flow traces showing the first inhalation after odor onset for early and late trials. Dotted gray line indicates where flow = 0 for each sniff cycle shown. Right: heatmap showing change in inhalation duration as a function of sniff number since odor onset, sorted by ΔMID. (G) Example nasal flow traces during CS^+^ presentations for example “high motivation” (left) and “low motivation” mice (right), for an early (top) and late (bottom) trial. “Motivation” here refers to the number of licks during the odor stimulus (“anticipatory” licks). Licks are shown as blue ticks. Droplet indicates when mouse would receive reward. Note that sniff changes only occur for the “high motivation” mouse. (H) ΔMID (averaged for each cell [n = 18] across CS^+^ and CS^−^ stimuli) as a function of the mean number of anticipatory licks in late trials (calculated from CS^+^ trials only). (I) ΔMID across learning (averaged for each cell across CS^+^ and CS^−^ stimuli, n = 18) as a function of the reaction time calculated from divergent lick patterns (Figure S2A).

What are the mechanisms underlying these response changes? Odors are acquired from the environment through sniffing behavior, which is subject to complex contextual modulation (Kepecs et al., 2007, Wachowiak, 2011, Wesson et al., 2009). Since sniffing controls input to the OB, sniff changes could potentially explain the learning-related changes in odor responses. To analyze whether sniffing changed across learning within the short 500 ms time window of the odor stimulus, we measured nasal flow using an external sensor and quantified the mean inhalation duration (MID) of all inhalations completed within this time window (Figure 3A). We chose MID because external measurement of sniffing does not give reliable data about sniff amplitudes (the naris can move relative to the sensor) and because MID correlates with sniff frequency and inhalation slope on a sniff-by-sniff basis—thus, changes in MID also reflect changes in these parameters (Jordan et al., 2017). We again compared five early and five late trials to quantify the change in MID across learning (ΔMID). This revealed significant changes in sniff behavior, e.g., the emergence of faster, sharper inhalations (reduced MID; Figure 3B). Reductions in MID mirrored increases in sniffing frequency and peak inhalation slope (Figures 3C, S8A, and S8B) and are thus indicative of faster sniffing. Across all cell-odor pairs, 26% underwent significant changes in MID during learning (p < 0.01, unpaired t tests), while only 11% underwent such significant changes in passively exposed mice (Figure 3D). Learning mice displayed significantly more variation in ΔMID (learning, SD = 24 ms; passive, SD = 9 ms; p = 3 × 10^−8^, Bartlett test; Figure 3E) and a significantly larger proportion of reductions in MID exceeding 20 ms (learning, 26%; passive, 2%; p < 0.01, bootstrapping; Figure 3E). Thus, the development of rapid sniffing was specific to learning mice. Note that, while many mice underwent significant changes in sniff strategy across learning, MID was already significantly lower for learning mice than for passive mice in early trials (p = 0.001, rank-sum test; Figure 3D), indicating potential retention of sniff strategies learned during pre-training.

Similar to the MTC response changes occurring across learning, changes in MID were correlated between CS^+^ and CS^−^ odors (Figure S8C) and occurred in the first inhalation after odor onset (Figures 3F and S8D). This suggests that MID changes in learning mice reflect a change in active sampling strategy rather than changes concomitant with reward anticipation or licking response.

What causes the variance in sniff changes across mice? Response vigor has previously been used as a measure of motivation levels in mice (Berditchevskaia et al., 2016). We calculated the amount of anticipatory licking for each mouse as a measure of motivation for the task. Anticipatory licks were defined as licks occurring during the CS^+^ odor stimulus (0.5–2 s after odor onset) during criterion performance in late trials. Since the relevant response window is the 1 s after odor offset, these licks do not contribute to the gaining of reward. Some mice showed a high rate of anticipatory licking, while others only licked during the designated response period (Figure 3G). Reductions in MID were significantly correlated with higher frequency anticipatory licking (R^2^ = 0.54, p = 4 × 10^−4^; Figure 3H). Since this correlation existed for CS^+^ and CS^−^ data alone (Figure S8G), these associations were not due to motor effects of licking. Consistent with previous data (Wesson et al., 2008), reduced MID was also significantly associated with shorter reaction time (Figure 3I; R^2^ = 0.23, p = 0.04). Overall, a multiple linear regression model with terms for the vapor pressure of each odor, an interaction term between the vapor pressure of the two odors, the anticipatory lick rate, and MID in early trials explained most of the variation in ΔMID (CS^+^, R^2^ = 0.71, p = 0.002; CS^−^, R^2^ = 0.76, p = 0.001), indicating that several contextual variables influence the tailoring of sniff strategy to the behavioral task.

Thus, mice developed active sampling strategies across the learning session, dependent on motivational state.

### Positive Response Changes Are Tightly Linked to Changes in Active Sampling

**Figure 4:**
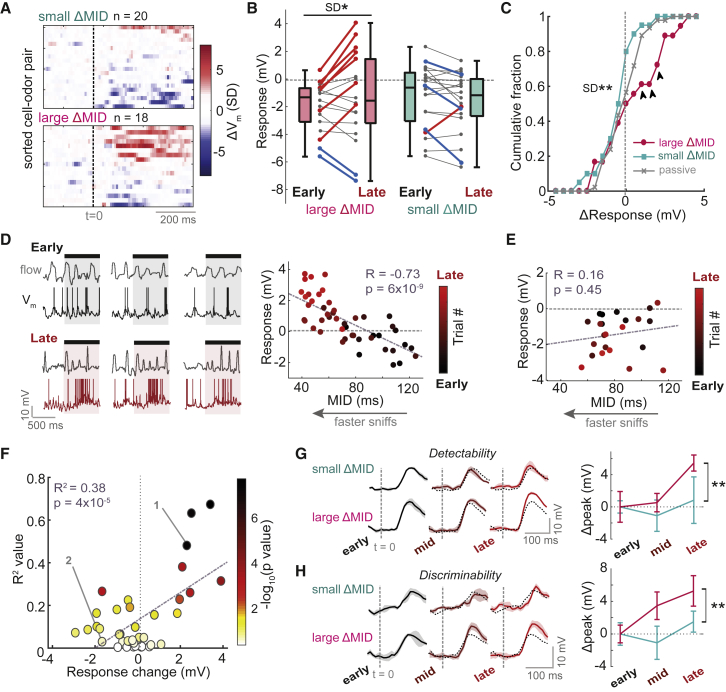
Positive Response Changes Are Tightly Linked to Changes in Active Sampling All data are from the learning dataset. (A) Response change heatmaps (late-early average V_m_ response) normalized by baseline SD, for small MID change (|ΔMID| <20 ms) and large MID change (|ΔMID| >20 ms). (B) Plot of early and late V_m_ responses for cell-odor pairs with large and small ΔMID separately. Thick red and blue lines indicate significant positive and negative changes, respectively (p < 0.01). (C) Cumulative histograms of V_m_ response changes. Black arrowheads indicate significant differences between large ΔMID and both small ΔMID and passive histograms (STAR Methods). Large ΔMID, SD = 1.9 mV, n = 18; small ΔMID, SD = 1.1 mV; p = 0.002, Bartlett test. (D) Left: example nasal flow and V_m_ traces for early and late trials for a cell-odor pair undergoing significant increase in excitation across learning. Spikes have been clipped for display. Right: scatterplot between MID and V_m_ response across trials for this cell-odor pair. Points have been colored according to trial number. (E) As for (D), but for a cell undergoing a significant increase in inhibition across learning. (F) Scatterplot between the response change across learning, and the R^2^ value for correlations as in (D) and (E), colored according to the p value of the correlation. Labeled points 1 and 2 refer to examples in (D) and (E), respectively. (G) Left plots: Euclidean distance between population response vectors and baseline data (measure of response detectability; Figure S13) for cell-odor pairs recorded alongside large ΔMID and small ΔMID. Shaded area shows SD. Dashed gray line indicates odor onset. Dotted black plots show data for early trials for comparison. Right: plot to show change in peak detectability within the first 170 ms of the stimulus for early, mid-point, and late trials, relative to the mean for early trials. Error bars show SD. Two-way ANOVA; ΔMID, p = 4 × 10^−12^; time, p = 0.0003; interaction, p = 0.02. (H) As for (G), but for the Euclidean distance between population response vectors for CS^+^ and CS^−^ (measure of response discriminability). Large ΔMID is assigned to cells with >20 ms change for both CS^+^ and CS^−^ (n = 8). Small MID change is assigned to all other cells (n = 11). Two-way ANOVA; ΔMID, p = 2 × 10^−7^; time, p = 0.0003; interaction, p = 0.008.

We next wanted to test what impact the changes in active sampling (Figure 3) had on the response changes observed across learning (Figure 2). We first split the dataset according to MID change: large MID change (>20 ms absolute change between early and late trials, n = 18) and small MID change (<20 ms absolute change, n = 20). We chose 20 ms to constitute a “large” MID change since such changes are frequent in learning mice while rarely seen in passive mice (Figure 3E). Comparing response changes between early and late trials for each dataset revealed that positive changes were almost exclusively displayed alongside large MID change (Figure 4A). There was a significant increase in response variance for cell-odor pairs recorded alongside large MID change (early SD = 1.8 mV, late SD = 3.2 mV; p = 0.02, Bartlett test), but not for small MID change (early SD = 2.2 mV, late SD = 2.2 mV; p = 0.98, Bartlett test; Figure 4B). In particular, there were significantly more positive response changes (>1 mV) occurring alongside large MID change (39%) compared to small sniff change (5%) and passive exposure (11%; p < 0.01, bootstrapping; STAR Methods; Figure 4C), while response changes recorded alongside small MID change were indistinguishable from passive controls (p = 0.94, Bartlett test, n = 20 versus 46). Altogether, only cell-odor pairs recorded alongside large MID change displayed significantly larger response variance in late trials compared to passive controls (small ΔMID versus passive, p = 0.50; large ΔMID versus passive, p = 0.02, Brown-Forsythe test).

To test the strength of associations between V_m_ response and active sampling further, we correlated MID and V_m_ response across trials for each cell-odor pair. For cells undergoing positive response changes across learning, this resulted in robust correlations (e.g., Figure 4D), while those undergoing increases in inhibition showed no such relationships (e.g., Figure 4E). This effect across the dataset resulted in a significant positive relationship between the response changes occurring across learning and the R^2^ of the correlation between MID and response across trials (R^2^ = 0.38, p = 4 × 10^−5^, n = 42; Figure 4F). These results were consistent even when considering CS^+^ or CS^−^ cell-odor pairs alone (Figures S7F and S7G). Most cell-odor pairs showed more positive odor responses as MID decreased, as in Figure 4D, while the opposite trend was less common (Figures S9A–S9C).

How did changes in active sampling impact changes in odor representation across the dataset? To test this, we split the dataset according to MID change as before (Figures 4A–4C), constructed population response vectors from each dataset, and calculated the Euclidean distance between odor response vectors and baseline data (STAR Methods; Figure S13). This provides a measure of response detectability. To look at a timescale relevant to decision making, we analyzed the peak detectability occurring within the first 170 ms after odor onset. We found that an increase in peak detectability occurred in the large ΔMID dataset, which was significantly greater than for the small ΔMID dataset (large ΔMID, late Δpeak = 5.4 ± 1.0 mV; small ΔMID, late Δpeak = 0.8 ± 2.9 mV; Cohen’s *d* = 1.5; p = 0.009, unpaired t test, n = 5; Figure 4G). We then used the Euclidean distances calculated between response vectors for CS^+^ and CS^−^ stimuli as a measure of stimulus discriminability. Again, the increase in peak discriminability was significantly greater for the large ΔMID dataset (large ΔMID, late Δpeak = 5.3 ± 1.9 mV; small ΔMID, late Δpeak = 1.5 ± 1.2 mV; Cohen’s *d* = 1.5; p = 0.006, unpaired t test, n = 5; Figure 4H). Consistent results were found for changes in spike rate responses despite their more variable nature at such short timescales (Figures S6F and S6G), indicating that these representational changes are relevant for OB output. No such changes in representation occurred in passive mice (Figures S9D and S9E). Thus, sniff changes coincided with the enhancement of odor representation.

### Active Sampling and Associated Response Changes Are Dynamically Linked to Task Engagement

**Figure 5:**
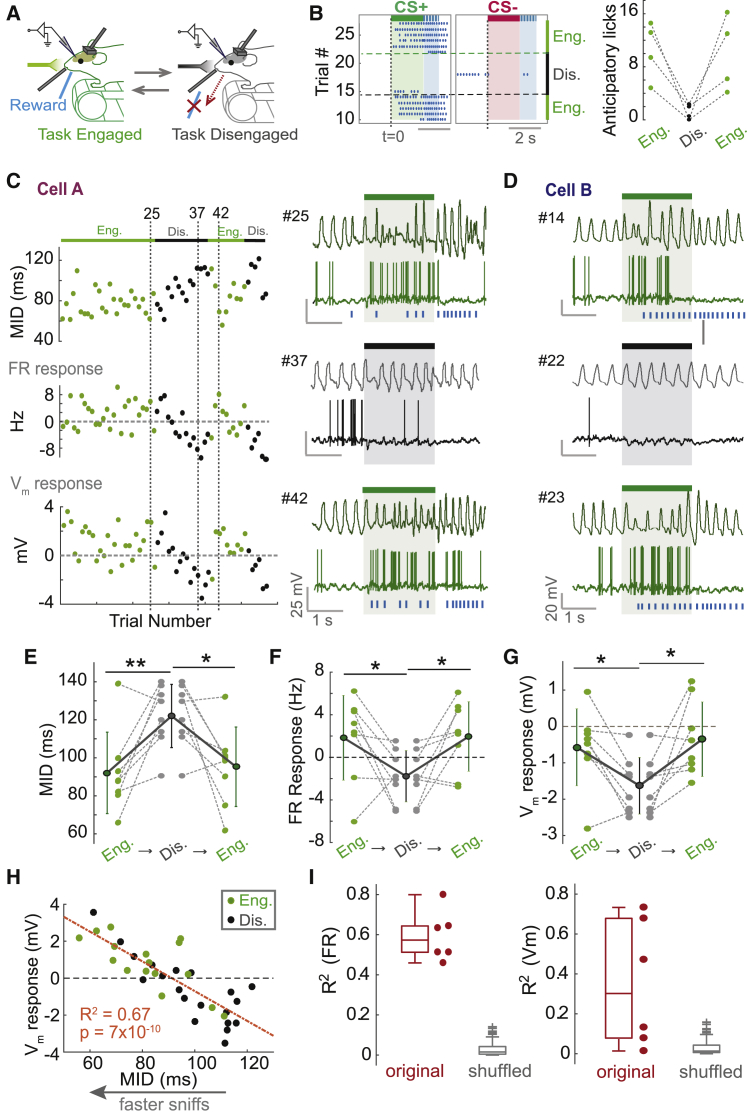
Active Sampling and Associated Response Changes Are Dynamically Linked to Task Engagement For all panels, green, task engaged (Eng.); black, task disengaged (Dis.). (A) Experimental paradigm for switches in task engagement. (B) Left: lick raster across task switches for CS^+^ and CS^−^ stimuli for an example mouse. Blue-shaded region shows 1 s response window after odor offset. Right: plot showing changes in anticipatory lick rate between engaged and disengaged trials for the CS^+^ for each mouse. (C) Top: plot showing MID across trials for an example cell-odor pair. Middle: corresponding plot to show odor FR response for the same cell-odor pair across trials. Bottom: corresponding plot to show V_m_ responses (after spike subtraction) across trials. Traces on the right show example nasal flow and V_m_ traces for trials corresponding to those indicated by dotted lines on the left plots. (D) Example traces for a different cell-odor pair during task engagement (top trace, trial #14), disengagement (middle trace, trial #22), and re-engagement (bottom trace, trial #23). Note that response valence can change within a single trial depending on task engagement. (E) For all eight cell-odor pairs, changes in MID between task engagement, disengagement, and re-engagement (asterisks denote result of paired t tests). Error bars show SD. (F) As for (E), but for changes in 2 s FR responses. (G) As for (E), but for changes in 500 ms V_m_ responses. (H) Scatterplot of MID versus V_m_ response across trials for an example cell-odor pair. (I) Left: boxplots to show R^2^ values (as for example in H) for all six FR responses showing significant changes between engagement shifts, alongside those for shuffled data. Right: as for left, but for V_m_ responses.

We next wanted to investigate the effect of dynamic changes in behavioral state on the changes in active sampling and odor responses observed. To do this, we recorded from eight cell-odor pairs in a new cohort of mice that were trained to criterion on the task prior to recording. If rapid sniffing is indeed an active strategy for odor acquisition during behavior, we would expect the strategy to disappear if the task comes to an end (i.e., transition to passive odor exposure) and re-emerge when the task reinitiates. To test this, we implemented a paradigm in which task engagement could be reversibly changed by physically removing and re-introducing the water reward spout (Figure 5A), resulting in rapid switches between olfactory behavior and passive exposure as indicated by anticipatory licking responses (Figure 5B). As predicted, animals robustly adapted their sniffing strategy upon elimination of the licking response after removal of the reward port (Figures 5C, top plot, and 5E), with MID increasing (slower sniffing), while reintroduction of the reward port rapidly restored fast sniff behavior (reduced MID).

If fast active sniffing determines positive response change as predicted from learning mice (Figure 4), we would expect positive changes to occur alongside the rapid sniffing strategy. We found that responses could change robustly and reversibly between task engagement, disengagement, and re-engagement, with some examples showing dramatic and reversible switches between excitation and inhibition (Figures 5C, 5D, and S10A–S10D). Consistent with the learning-related changes, positive response changes always occurred alongside reduced MID (Figures 5E–5G) and were again tightly linked to MID on a trial-by-trial basis (Figures 5H and 5I). Strikingly, response changes could occur within only a single trial upon recognition of task re-engagement (Figures 5D and S10E), emphasizing the dynamic nature with which changes in active sampling state influence neural responses.

### Odor Response Changes Associated with Active Sniffing Are Dependent on Behavioral State

**Figure 6:**
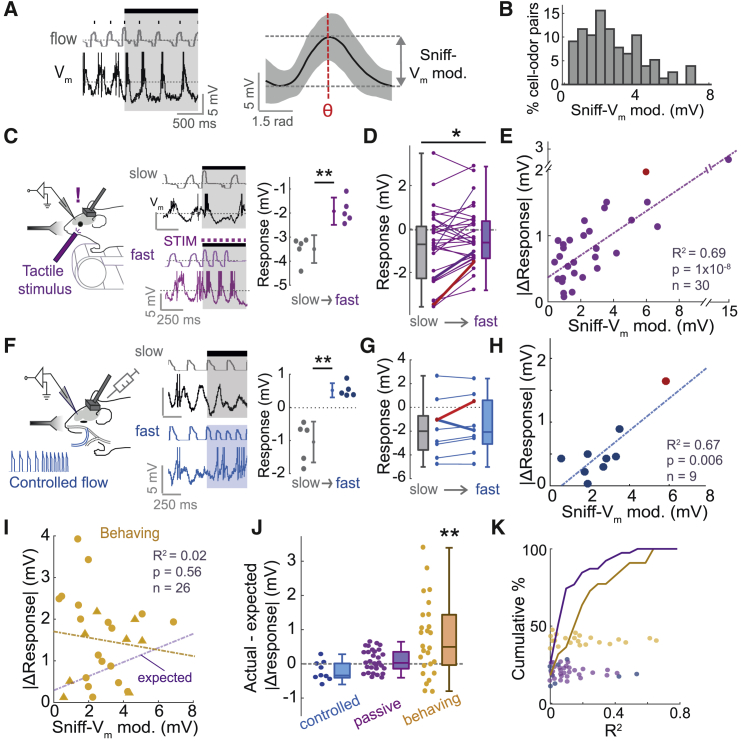
Response Changes Associated with Sniff Changes Are Dependent on Behavioral State (A) Left: example V_m_ trace from a cell (recorded in a passive mouse) showing strong modulation by the sniff cycle. Right: average V_m_ as a function of sniff phase during the odor stimulus for this example cell. Shaded area shows SD. Sniff-V_m_ modulation amplitude is calculated as the difference between the minimal V_m_ and the value of V_m_ at the preferred phase, as shown (Figure S11B). (B) Histogram of sniff-V_m_ modulation amplitudes across cell-odor pairs recorded in learning mice (n = 38) and passive (n = 42) mice. (C) Left: experimental setup for tactile stimulation of passive mice. Middle traces show nasal flow and V_m_ from an example cell: top, control trial (slow sniffing); bottom, tactile stimulus trial (fast sniffing). Right plot shows mean V_m_ responses for five slow and five fast sniff trials for this example cell. Error bars show SD. (D) Plot to show mean responses averaged across 5 “slow” and 5 “fast” sniff trials for 30 cell-odor pairs. Red line indicates example in (C). (E) Scatterplot of absolute response change (between slow and fast sniff trials) versus sniff-V_m_ modulation amplitude during the odor. Red point shows example in (C). (F) Experimental set up for controlled nasal flow via double tracheotomy in anesthetized mice. Example traces show nasal flow and V_m_ trace for one cell: top = slow sniff trial (3.3 Hz); bottom = fast sniff trial (6.6 Hz). Right plot shows mean V_m_ responses for five slow and five fast sniff trials for this example cell. Error bars show SD. (G) As for (D), but for “controlled-flow” anesthetized mice (n = 9 cells). (H) As for (E), but for “controlled-flow” anesthetized mice (n = 9 cells). (I) As for (E) and (H), but for cell-odor pairs recorded during learning (circles) or task engagement (triangles), where |ΔMID| >20 ms. Yellow line indicates linear regression for these data, while purple line indicates that for pooled data from (E) and (H), which was used to calculated “expected” |Δresponse| values. (J) Plot comparing deviation of response changes from expected values (see I), for controlled-flow, passive, and behaving mice. (K) Cumulative histograms to show R^2^ values for correlations between MID and V_m_ response calculated across trials for each cell odor pair undergoing large MID change in behaving mice (n = 26, gold line and data points) and pooled data (purple line) from controlled-flow (blue data points, n = 9) and passive mice (purple data points, n = 30). Behaving, median = 0.16, IQR = 0.03–0.27; passive and controlled flow, median = 0.07, IQR = 0.02 to 0.15; p = 0.07; rank-sum test; p = 0.09, Brown-Forsythe test.

We next wanted to assess to what degree the response changes during active sniffing arise from a purely feedforward mechanism. MTC activity is patterned by the sniff cycle in anesthetized mice, giving rise to sniff-coupling of membrane potential (Adrian, 1950, Cang and Isaacson, 2003, Fukunaga et al., 2012, Fukunaga et al., 2014, Macrides and Chorover, 1972, Margrie and Schaefer, 2003, Schaefer et al., 2006), which we will refer to as “sniff-V_m_ modulation.” Preventing rhythmic nasal flow results in an abolition of sniff-V_m_ modulation (Courtiol et al., 2011, Margrie and Schaefer, 2003, Schaefer et al., 2006; Figure S11A), indicating that they arise from feedforward, sniff-locked sensory input from olfactory sensory neurons (OSNs). To estimate the amount of sniff-locked input in awake mice, we calculated the amplitude of sniff-V_m_ modulation for each cell-odor pair (Figures 6A and S11B), revealing a wide range of amplitudes up to 7 mV during odor stimulation (Figure 6B). It is thus possible that rapid sniffing can evoke changes in response via changes in the sniff-locked input pattern from OSNs.

If response changes during active sniffing arise purely from feedforward input, then similar sniff changes should evoke comparable response changes even in absence of an olfactory task. We found that unexpected tactile stimulation briefly increased sniff rates in passive mice (Figure 6C), quantitatively reproducing (and even exceeding) the sniff changes seen during learning (Figure S11C). When paired with odor delivery, this resulted in a variety of largely positive odor response changes (ΔV_m_ response = 0.45 ± 0.96 mV, p = 0.01, paired t test, n = 30; Figure 6D). If these are mediated by changes in sniff-locked input, we may expect the changes to correlate with the degree to which the response is sniff coupled. Indeed, the response changes were strongly correlated with the amplitude of sniff-V_m_ modulation, such that highly sniff-locked cells underwent the largest changes when sniffing was altered (R^2^ = 0.69, p = 1 × 10^−8^, n = 30; Figure 6E). These response changes are unlikely to be due to changes in arousal or from somatosensory input, since they were similarly present in anesthetized mice, where using a double tracheotomy the frequency of artificial sniffing (nasal flow) could be controlled independently of free tracheal breathing (Figures 6F and 6G). Response changes in these “controlled-flow” mice were also significantly correlated with sniff-V_m_ modulation amplitude (R^2^ = 0.67, p = 0.006, n = 9; Figure 6H). Thus, in absence of olfactory behavior, evoking sniff changes results in response changes that depend on the amount of sniff-locked input to the cell.

We next wanted to assess whether this was the case for response changes during active sniffing in behaving mice. We thus pooled response changes from learning and task-engaged mice in which MID underwent a change exceeding 20 ms, yielding 26 “behaving” cell-odor pairs in total. In contrast to passive and controlled-flow mice, there was no correlation between response changes and sniff-V_m_ modulation amplitudes (R^2^ = 0.02, p = 0.56, n = 26; Figure 6I). Using the linear model resulting from the correlation for pooled data from passive and controlled-flow mice (ΔV_ex_ = 0.17^∗^*T* + 0.29 mV, where ΔV_ex_ = expected absolute V_m_ response change and *T* = sniff-V_m_ modulation amplitude), we generated “expected” values for V_m_ response change based on the sniff-V_m_ modulation amplitude of each cell odor pair. Response changes in behaving mice significantly exceeded those expected based on their sniff-V_m_ modulation amplitude (actual-expected difference = 0.72 ± 1.1 mV, n = 26, p = 0.002, paired t test; Figure 6J), while (by construction) this was not the case for cells from passive (actual-expected difference = 0.08 ± 0.33 mV, p = 0.16, paired t test, n = 30 cell-odor pairs) or controlled-flow mice (actual-expected difference = −0.21 ± 0.25 mV, p = 0.05, paired t test, n = 9 cell-odor pairs). This effect could not be explained by variance in ΔMID, since the result was the same when we used a model including a term for the magnitude of ΔMID (Figures S11D and S11E).

While this suggests that sniff-evoked response changes in behaving mice exceed those expected based purely on sniff-locked feedforward input, this does not mean that such response changes are any less linked to the sampling behavior of the animal. When comparing R^2^ values for the correlation between MID and V_m_ response across trials for each cell-odor pair, we found that V_m_ responses in behaving mice showed slightly more robust correlations with MID on a trial-by-trial basis than in passive or controlled-flow mice (Figure 6K).

Thus, in odor-attentive mice, response changes during active sniffing exceed those expected based only on the feedforward input to the cell, indicating a potential top-down component contributing to these response changes.

### Effect of Fast Sniffing in Absence of Applied Odor Depends on Feedforward Input in Learning and Passive Mice

Since the baseline activity of MTCs is widely modulated by the sniff cycle (Cang and Isaacson, 2003, Fukunaga et al., 2012, Fukunaga et al., 2014, Macrides and Chorover, 1972; Figure S4F), it is likely that sniff changes would cause activity changes even in absence of applied odor stimulus. We thus wanted to test whether the enhancement of response change during active sniffing in behaving mice (Figures 6I and 6J) was restricted to the odor sampling period.

**Figure 7:**
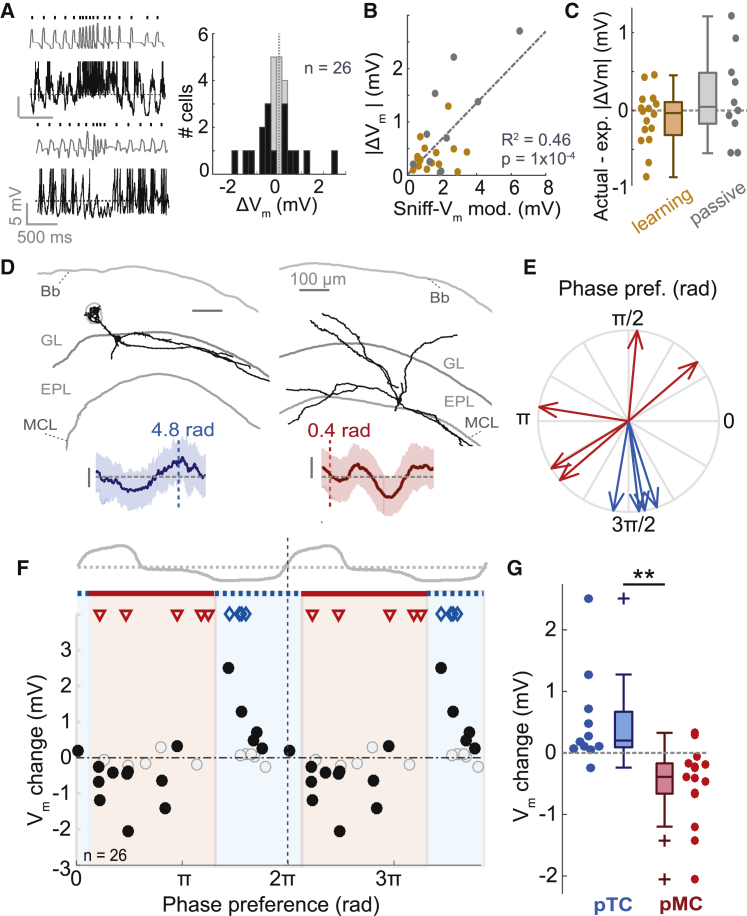
Response Changes in Absence of Applied Odor Are Dependent on Sniff-Locked Input (A) Left: example traces showing spontaneous fast sniff bouts during inter-trial interval and concurrent V_m_ traces. Spikes are clipped for display. Right: histogram to show distribution of V_m_ changes during spontaneous rapid sniffs (>5 Hz) for 26 MTCs in which there were >20 fast sniffs. Black bars indicate cases showing a significant change in V_m_. (B) Scatterplot of absolute V_m_ change (between slow and fast sniffs) against sniff-V_m_ modulation amplitude. Gray dots show data from passive mice (n = 10); gold dots show data from behaving mice (n = 16). (C) Plot to show differences between actual and expected V_m_ change (expected V_m_ change is calculated based on sniff-V_m_ modulation amplitudes using linear regression in B). (D) Morphologies of a reconstructed TC (left) and MC (right), with mean V_m_ as a function of sniff phase shown below (shaded area = SD). Phase preferences are indicated with dotted lines. Bb, brain border; GL, glomerular layer; EPL, external plexiform layer; MCL, mitral cell layer. (E) Phase plot to show preferences of five reconstructed MCs (red) and four reconstructed TCs (blue). 0/2π radians = inhalation onset. (F) V_m_ change (between fast and slow sniffs) as a function of the sniff-phase preference of the cell. Black filled dots show significant V_m_ changes. Red-shaded region shows phases that best encompass hyperpolarizing cells (putative MCs), and remaining blue region best encompasses depolarizing cells (putative TCs). Symbols show phase preferences of morphologically recovered cells: red triangles, MCs; blue diamonds, TCs. (G) Comparison of V_m_ change due to fast sniffing for putative TCs and MCs defined by the phase boundaries shown in (F). pMCs, median ΔV_m_ = −0.39 mV, IQR = −0.66 to −0.17 mV, n = 16; pTCs, median ΔV_m_ = 0.19 mV, IQR = 0.08 to 0.66 mV, n = 11; p = 9 × 10^−4^, rank-sum test.

To examine this, we made use of spontaneous bouts of rapid (>5 Hz) sniffing that occur in awake mice during the ITI—i.e., in absence of applied odor. Consistent with previous data (Kato et al., 2013), overt depolarization or hyperpolarization would occur in some cells coinciding with such rapid sniff bouts (Figure 7A). Quantifying the change in mean V_m_ during fast sniffing across 26 MTCs revealed that almost two-thirds showed significant changes, with 7 depolarizing and 9 hyperpolarizing (p < 0.05, bootstrapping; STAR Methods; Figure 7A). These changes correlated well with the sniff-V_m_ modulation amplitudes of each cell (R^2^ = 0.46, p = 0.001, n = 26; Figure 7B), indicating that these changes are again likely the result of changes in feedforward input. Comparing the actual V_m_ change to that expected based on the linear regression model in Figure 7B (ΔV_ex_ = 0.31^∗^*T* + 0.01 mV, where ΔV_ex_ = expected absolute V_m_ change and *T* = sniff-V_m_ modulation amplitude) showed that actual and expected V_m_ changes did not significantly differ for either passive (0.17 ± 0.57 mV, p = 0.37, paired t test, n = 10) or behaving cell-odor pairs (−0.11 ± 0.36 mV, p = 0.25, paired t test, n = 16; Figure 7C), and the actual – expected difference was indistinguishable between passive and behaving datasets (p = 0.14, unpaired t test; Cohen’s *d* = 0.25; p = 0.1, Bartlett test). Altogether, this indicates that enhanced response change during rapid sniffing in a behaving animal only occurs during the odor sampling period.

Since cells could either depolarize or hyperpolarize during fast sniffing, we sought to determine whether the sign of response change was also predictable from sniff-locking properties. Evidence from anesthetized mice suggests that MCs are driven by feedforward inhibition and lock to inhalation, while TCs are driven by feedforward excitation and lock to exhalation (Fukunaga et al., 2012, Fukunaga et al., 2014). To test this in awake mice, we recovered 9 morphologies of MTCs (e.g., Figure 7D) and identified them as MCs (n = 5) or TCs (n = 4). Congruent with the previous data, the two cell types had membrane potentials that locked to distinct phases of the sniff cycle: MCs locked to inhalation, while TCs locked to exhalation (Figure 7E). We next examined the relationship between phase preference and the effect of fast sniffing across the full sample of cells. The sign of the V_m_ change during fast sniffs was strongly related to the sniff-phase preference of the cell (Figure 7F), with inhalation-locked cells hyperpolarizing and exhalation-locked cells depolarizing. We calculated the phase boundaries for best separation of hyperpolarizing and depolarizing cells (as shown in Figure 7F; STAR Methods). Cells within the inhalation boundaries (0.39–4.11 rad; putative MCs) showed significantly more hyperpolarizing effects of fast sniffing than those within the exhalation boundaries (putative TCs; Figure 7G). Indeed, the phase preferences of morphologically identified MCs and TCs conformed to these boundaries (Figure 7F, red triangles and blue diamonds).

Thus, in absence of applied odor, the effect of fast sniffing on V_m_ is predicted by the sniff-driven input of the cell regardless of behavioral state, indicating that enhanced response changes during active sniffing in behaving mice only occur during odor sampling.

### Tufted Cells Show More Highly Correlated Changes Than Mitral Cells

Since previous work has suggested that both learning and neuromodulatory activity may have divergent effects on MC and TC odor responses (Kapoor et al., 2016, Yamada et al., 2017), we next wanted to compare response changes for the two groups of cells. To this end, we used the phase preference boundaries (Figure 7F) to designate putative mitral (pMC) and tufted cell (pTC) phenotype. Consistent with the idea that these boundaries can separate TCs and MCs, mean FR responses to odors in pTCs showed a significant tendency toward strong excitation compared to pMCs (Figure S12), congruent with previous data (Fukunaga et al., 2012, Nagayama et al., 2004).

**Figure 8:**
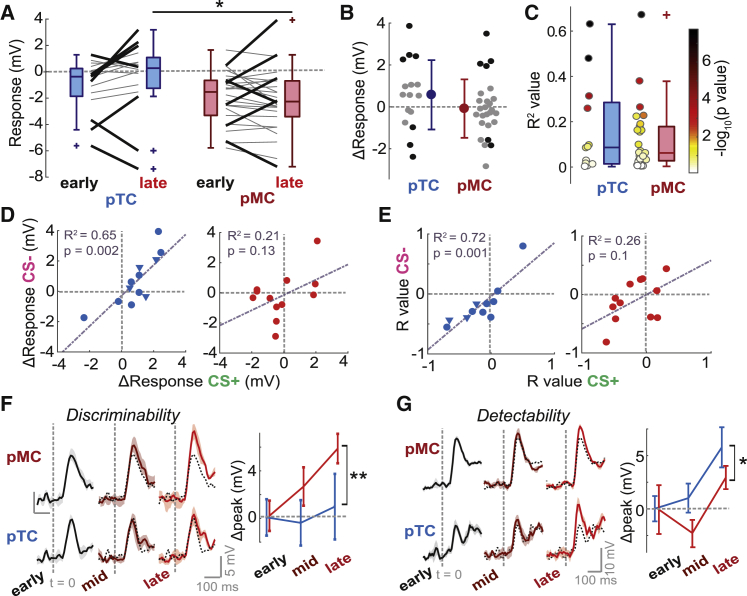
Tufted Cells Show More Highly Correlated Changes Than Mitral Cells All data are from the learning dataset unless indicated. (A) Plot between early and late V_m_ responses for pTCs (n = 16 cell-odor pairs) and pMCs (n = 26 cell-odor pairs) separately. Thick black lines show significant changes (p < 0.01, unpaired t tests). Early, pTCs, −1.1 ± 1.9 mV; pMCs, −1.8 ± 2 mV; p = 0.26, unpaired t test; late, pTCs, median = 0.3 mV, IQR = −1.3 to 1.1 mV; pMCs, median = −2.1 mV, IQR = −3.2 to 0.5 mV; p = 0.01, rank-sum test. (B) Comparison of response changes (late-early) for pTCs and pMCs. Black dots show significant changes (p < 0.01, unpaired t test). (C) Comparison of R^2^ values for correlations between MID and V_m_ response across trials for pTCs and pMCs. Color shows p value of the correlation (−log_10_). pTCs, median R^2^ = 0.09, IQR = 0.01 to 0.29; pMCs, median R^2^ = 0.06, IQR = 0.03 to 0.18; p = 0.88, rank-sum test; p = 0.35, Brown-Forsythe test. (D) Scatterplots between response changes for CS^+^ and CS^−^ odors. Triangles show data from task-engagement recordings (as in Figure 5; response change = engaged – disengaged). Circles are from learning data. (E) As for (D), but for R values from the correlations between MID and V_m_ response across trials for each cell. (F) Left: Euclidean distance between population response vectors for CS^+^ and CS^−^ (a measure of discriminability) for pTCs and pMCs independently. Shaded area shows SD. Black dotted plot shows data for early trials for comparison. Right: plot showing change in peak discriminability (relative to mean for early trials) in the first 170 ms of the stimulus for early, mid-point, and late trials for pTCs (blue) and pMCs (red). Error bars show SD across five trial subsets. Two-way ANOVA; cell type, p = 8 × 10^−6^; time, p = 0.002; interaction, p = 0.02. (G) As for (F), but for Euclidean distances between odor response and baseline data (a measure of detectability). Two-way ANOVA; cell type, p = 4 × 10^−18^; time, p = 3 × 10^−7^; interaction, p = 0.06.

The distribution of V_m_ responses in early trials did not significantly differ between pMCs and pTCs, though pTC responses tended to show less inhibition (p = 0.26, unpaired t test; Figure 8A). Response changes across learning for putative MCs and TCs also did not significantly differ in terms of mean or variance but tended to be more positive for pTCs (pTCs, 0.64 ± 1.7 mV; pMCs, −0.14 ± 1.4 mV; p = 0.1, unpaired t test, Cohen’s *d* = 0.52; p = 0.46, Bartlett test; Figure 8B). This meant that, in late trials, pTCs showed significantly more positive responses compared to pMCs (p = 0.01, rank-sum test; Figure 8A), consistent with previous findings that TCs receive less inhibition than MCs (Christie et al., 2001). Comparing the R^2^ values for the correlations between MID and V_m_ response across trials also indicated that, in general, pMCs and pTCs do not show differing effects of sniffing on responses (Figure 8C).

We next compared response changes for CS^+^ and CS^−^ stimuli for pMCs and pTCs individually. For pTCs, response changes for the two stimuli were strongly correlated (R^2^ = 0.65, p = 0.002, n = 12 cells), whereas this was weaker for pMCs (R^2^ = 0.21, p = 0.13, n = 13 cells; Figure 8D). The same difference was seen when looking at the R values for correlations between MID and V_m_ response across trials (Figure 8E). Given this difference, we next compared the changes in response discriminability across learning. Using the Euclidean distance between population response vectors for CS^+^ and CS^−^ stimuli, we found that pMCs underwent a significantly larger increase in peak discriminability by late trials compared to pTCs (pMCs, late Δpeak = 4.8 ± 1.0 mV; pTCs, late Δpeak = 0.7 ± 2.4 mV; Cohen’s *d* = 1.5; p = 0.009, unpaired t test, n = 5; Figure 8F). Both cell types contributed to increased detectability of the stimulus across learning, though this was more pronounced for pTCs than pMCs (pMCs, late Δpeak = 2.9 ± 1.1 mV; pTCs, late Δpeak = 5.6 ± 1.8 mV; Cohen’s *d* = −1.4; p = 0.02, unpaired t test, n = 5; Figure 8G).

Thus, changes in active sniffing are associated with cell-type-specific effects on odor representation.

## Discussion

Active sampling behavior is a fundamental feature of sensory information acquisition. Theoretical and psychophysical evidence has driven hypotheses that active sampling strategies during behavior may be used to optimize sensory information flow (Ahissar and Assa, 2016, Laing, 1983, Yang et al., 2016). Here, we provide new evidence for modulation of early sensory processing in the OB during active sampling epochs, which serves to enhance early odor representation.

### Sniffing Strategy Is Highly Context Dependent

Rodents alter their sniffing pattern in many contexts (Wachowiak, 2011), both in the presence (Kepecs et al., 2007, Roland et al., 2016, Wesson et al., 2009, Youngentob et al., 1987) and absence (Bramble and Carrier, 1983, Ikemoto and Panksepp, 1994, Wesson et al., 2008) of a task-relevant odor stimulus. Consistent with previous reports (Wesson et al., 2009), we find wide variance in sniff strategy across mice (Figure 3D), a large portion of which can be explained by motivational state (Figure 3H). Further taking into account other variables (the sniff strategy already being displayed in early trials and the vapor pressures of the odors being discriminated) can altogether explain more than 70% of the variation. Thus, active sampling strategies are highly context dependent and appear to be a function of (1) previous learning, (2) the properties of the odor pair being discriminated, and (3) the motivational level of the mouse.

The active sniffing strategy we describe here is characterized by a short bout of fast sniffs (around 3–4 sniff cycles), which occur prior to lick onset (Figure S8E), while previous work indicates that mice can perform such tasks within a single sniff cycle (Abraham et al., 2004, Abraham et al., 2010, Resulaj and Rinberg, 2015, Uchida and Mainen, 2003). During criterion performance, inhalation durations were perfectly correlated between the CS^+^ and CS^−^ in the first inhalation after odor onset and remained significantly (though less robustly) correlated for the second and third sniff cycle (Figure S8F), indicating that, while mice were certainly capable of performing the task in one sniff cycle, some may have taken a second or third sniff into account. Since sniff changes depended to some degree on the odor pair being discriminated, and previous work has shown that more difficult discriminations can increase reaction times (Abraham et al., 2004, Rinberg et al., 2006b), the persistence of sniffing over several sniff cycles could be due to the nature of the odor pair being discriminated, and because responding as fast as perceptually possible does not benefit the mouse in our task structure.

### Sniff Change as a Mechanism for Context-Dependent OB Responses

Previous studies into the effects of sniff variance on OB activity have often used controlled flow in tracheotomized preparations (Carey and Wachowiak, 2011, Cenier et al., 2013, Courtiol et al., 2011, Oka et al., 2009), or natural sniffing during passive exposure (Blauvelt et al., 2013, Cenier et al., 2013, Shusterman et al., 2017), while fewer cases have looked at OSN activity during task-engaged sniffing (Wesson et al., 2009, Verhagen et al., 2007). Altogether these show various effects of fast sniffing on odor responses in OSNs and MTCs: onset latency is reduced, while both response amplitude and patterning of activity by the sniff cycle are attenuated. Here, we further show that in absence of olfactory behavior, sniff changes will alter membrane potentials depending on the amount of feedforward sniff-locked input received by the cell (Figures 6E and 6H).

We advance these findings by observing changes in MTC responses during the development of active sniffing strategies within an odor task. We show that active sniff change is associated with a profound effect on a wide range of cells independent of their sniff-locking properties (Figures 6I and 6J), and that this effect is characterized largely by increased excitation to the odor (Figures 4 and 5) and enhances representation of odor stimuli in a cell-type-specific manner (Figures 4G, 4H, 8F, and 8G). This enhancement of odor representation could explain the improvement in discrimination performance (Kepecs et al., 2007) and faster reaction times (Figure 3I; Wesson et al., 2008) of mice displaying active sniffing strategies.

While context-dependent responses are described in a large number of studies on MTC activity (Chu et al., 2016, Fuentes et al., 2008, Kay and Laurent, 1999, Pager et al., 1972, Rinberg et al., 2006b, Yamada et al., 2017), it is notable that sniffing behavior is modulated by very similar contexts (Blanchard et al., 2001, Ikemoto and Panksepp, 1994, Kepecs et al., 2007, Wesson et al., 2008, Wesson et al., 2009). While their precise nature is dependent on behavioral context, changes in activity during sniff changes occur in all behavioral states (Figure 6). Changes in sniffing could therefore provide a common mechanistic basis for a number of different contextual modulations described in OB activity. However, we note that some variance in response change (such as the increases in inhibition during learning; Figures 4E and 4F) could not be explained by the sniff parameters we measured here. These could constitute a different form of plasticity altogether, or they may be explained by parameters of sniffing that we could not reliably measure using external sensors (such as amplitudes or inhalation volume).

### Putative Top-Down Influences during Active Sniffing

While response changes during fast sniffing were dependent on sniff-locked OSN input in passive and anesthetized mice, response changes far exceeded those predicted by the sniff-locked input in task-engaged/learning mice (Figure 6). This suggests the involvement of top-down centers that serve to coordinate sensory processing at the periphery with the active sampling state of the animal (Wachowiak, 2011). The evoked sniff changes in passive mice, and particularly the controlled sniff changes in anesthetized mice (Figure 6), causally address the feedforward component of response changes during active sniffing. However, the brain centers involved in the control of volitional active sniffing—which may provide a top-down influence on OB processing—are not yet well understood. Several neuromodulatory centers that project to the OB interact with respiratory control centers in the brainstem: raphe nuclei project to both to the OB and the respiratory brainstem, while the locus coeruleus projects to the OB and is modulated by the preBötzinger complex (Dugué and Mainen, 2009, Yackle et al., 2017). We find a cell-type specificity in the effect of active sampling on response changes, and neuromodulatory centers can have divergent effects on MCs and TCs (Kapoor et al., 2016). Thus, neuromodulators are a prime candidate to coordinate OB state with active sampling behavior. Future investigation will be required to address which centers are activated during active sampling, alongside their targets within the OB circuit.

Complex orchestration similarly occurs for other active sampling behaviors including whisking (Mitchinson et al., 2007) and eye movements (Rayner et al., 2007), with both behaviors affecting sensory cortical activity (Crochet and Petersen, 2006, McFarland et al., 2015). Whether and how directed adjustments to these sampling behaviors might also improve early stimulus representations remains to be seen.

In conclusion, we find that early sensory activity in the OB is overtly modulated during dynamic adjustments in active sniffing, yielding enhanced sensory representation for olfactory behavior.

## STAR★Methods

### Key Resources Table

**Table:** 

REAGENT or RESOURCE	SOURCE	IDENTIFIER
**Chemicals, Peptides, and Recombinant Proteins**		

Biocytin	Sigma-Aldrich	Cat #: B4261
3,3′-Diaminobenzidine tetrahydrochloride	Sigma-Aldrich	Cat #: D5905
Methyl salicylate	Sigma-Aldrich	Cat #: 76631
Cinnamaldehyde	Sigma-Aldrich	Cat #: C80687
1-nonanol	Sigma-Aldrich	Cat #: 131210
1-heptanol	Sigma-Aldrich	Cat #: H2805
0-ethylphenol	Sigma-Aldrich	Cat #: E44000
Acetophenone	Sigma-Aldrich	Cat #: 42163
Creosol	Sigma-Aldrich	Cat #: 302880
Ethyl heptanoate	Sigma-Aldrich	Cat #: 97587
Guaiacol	Sigma-Aldrich	Cat #: W253200
Valeric acid	Sigma-Aldrich	Cat #: 75054
(+)-carvone	Sigma-Aldrich	Cat #: 22070
2-phenyl ethanol	Sigma-Aldrich	Cat #: 77861
4-allyanisole	Sigma-Aldrich	Cat #: A29208
α-terpinene	Sigma-Aldrich	Cat #: 86473
Butyric acid	Sigma-Aldrich	Cat #: 19215
Limonene	Sigma-Aldrich	Cat #: 62118
Benzaldehyde	Sigma-Aldrich	Cat #: 09143
Eucalyptol	Sigma-Aldrich	Cat #: 29210

**Critical Commercial Assays**		

VECTASTAIN Elite ABC-HRP Kit	Vector Laboratories	Cat #: PK-6100

**Experimental Models: Organisms/Strains**		

Mouse: C57BL/6j	The Jackson Laboratory	Strain #: 000664

**Software and Algorithms**		

MATLAB	https://www.mathworks.com/products/matlab.html	RRID: SCR_001622
Spike2	http://ced.co.uk/downloads/latestsoftware	RRID: SCR_000903
R	http://www.r-project.org/	RRID: SCR_001905
LabView	http://www.ni.com/en-gb/shop/labview.html	RRID: SCR_001905

### Contact for Reagent and Resource Sharing

Further information and requests for resources and reagents should be directed to and will be fulfilled by the Lead Contact, Andreas T. Schaefer (andreas.schaefer@crick.ac.uk).

### Experimental Model and Subject Details

All animal experiments were approved by the local ethics panel of the Francis Crick Institute (previously National Institute of Medical Research) and UK Home Office under the Animals (Scientific Procedures) Act 1986. All mice used were C57BL/6 Jax males aged between 5 and 8 weeks obtained by in house breeding. All chemicals were obtained from Sigma-Aldrich (Missouri, USA).

### Method Details

#### Head-fixation

For surgical procedures, strict sterile technique was adhered to. Mice were anesthetized with isoflurane in oxygen (5% for induction, 1.5%–3% for maintenance), and received general analgesia (Carprofen, 5mg/kg s.c.) as well as local analgesia around the dorsal surface of the head (Levobupivicaine or Mepivicaine, 0.5% s.c.). A custom-made stainless steel headplate was affixed to the intraparietal and parietal skull plates with a combination of cyanoacrylate and dental cement, while a recording chamber was constructed upon the bone overlying the right OB using a plastic ring and dental cement. The chamber was filled with silicone (Quik-Cast - World Precision Instruments, Florida, USA) and sealed during the recovery and training periods prior to recordings. After 48 hours recovery, mice going on to passive experiments were head-fixed under very light isoflurane anesthezia (identical to the trained mice, see below) and allowed to awaken on a custom-made treadmill. Mice were allowed to accustom themselves to the treadmill in this initial 20 minute session, by the end of which mice showed no stress behavior and learned to walk and sit calmly on the treadmill. Mice going on to behavioral training underwent 2 days of additional water scheduling prior to head-fixation, and in the initial head-fixation session were additionally allowed access to abundant free rewards (diluted sweetened condensed milk) upon licking at the reward spout.

#### Go/No-Go behavior

The day following head-fixation habituation, mice undergoing Go/No-Go training progressed to two more days of pre-training for acquisition of the Go/No-Go task. On the first day mice were presented only the CS+ odor and were trained to acquire the ‘go’ licking pattern following odor offset via a delay classical conditioning procedure. Note that no measure was in place to prevent or punish licking behavior during the odor stimulus, and some mice would additionally lick during the odor stimulus prior to the allotted response time after odor offset (termed ‘anticipatory licking’). Following successful learning of this lick pattern, the next day mice were presented both the CS+ and CS- on a pseudorandom basis. Mice had to learn to respond to these odors differentially, learning to inhibit responses (‘no-go’ behavior) for the CS- to avoid a 5 s addition to the ITI. Only when mice had successfully demonstrated learning of this task (two consecutive 10-trial blocks of at least 80% correct responses) they were moved on to whole-cell recording procedures the next day (see below). After successful acquisition of a recording, mice were presented a novel pair of odor stimuli assigned each to CS+ or CS-, and had to learn the Go/No-Go behavior for these new stimuli. Criterion within a recording was considered one block of at least 80% correct performance. Learning of the task with the second pair of stimuli was always far more rapid than for the original acquisition (Figure 1B), well within whole-cell recording timescale in awake mice. For mice undergoing the task engagement/disengagement paradigm, acquisition of the task occurred prior to recording such that criterion performance was already achieved from the start of the recording. After 20-30 trials, the water port was manually moved away to disengage the task. Mice would continue to attempt to lick (as detected by infrared beam) for a variable number of trials before ‘giving up’ (i.e., 5 consecutive ‘miss’ trials), after which the port was returned. Often a free reward was used as a salient stimulus to the mouse that the task was re-engaged.

#### Odor delivery

Odor stimuli were delivered using a custom-made airflow dilution olfactometer with electronic dilution control. All odor stimuli were calibrated using a mini photoionization detector (miniPID, Aurora Scientific, Ontario, Canada) to form square-pulses of 1% concentration (relative to maximum recorded vapor-pressure in air; Figure S1). Odor stimuli used for initial go/no-go training purposes consisted of peppermint oil and almond oil - components that were not present in the odor mixtures later presented in recordings. For stimuli during whole-cell recordings, two were randomly selected from four potential odor mixtures (Figure S1), and for behaving mice randomly assigned to CS+ or CS-. Odor mixtures were comprised of four to six monomolecular odorants selected for their reported ability to activate dorsal glomeruli (Takahashi et al., 2004), grouped according to similarity of vapor pressure, and added to the mixture in an undiluted quantity inversely proportional to their relative vapor pressures (Figure S1). Odors were presented with a minimum ITI of 10 s. To minimize contamination, a high flow clean air stream was passed through the olfactometer manifolds during this time. Constant air-flow going to the animal was achieved using a final valve, minimizing any tactile component accompanying the odor stimulus.

#### Whole-cell recordings

Animals were anaesthetized under isoflurane as before, and recording chambers were re-opened. A 1-2 mm craniotomy and durectomy was made over the right OB. The craniotomy was then covered with a 0.5-1 mm layer of 4% low melting-point agar, which greatly contributed to the stability of recordings. This layer was removed and re-applied after every descent of a recording micropipette. The recording chamber was then filled with cortex buffer (125 mM NaCl, 5 mM KCl, 10 mM HEPES, 2 mM MgSO_4_, 2 mM CaCl_2_, 10 mM glucose), and the mice were transitioned to head-fixation and allowed 30 minutes to recover from anesthesia. After this time, behaving animals would demonstrate retention of go/no-go behavior acquired the day previously prior to attempt for a recording. Micropipettes were prepared with a resistance of 5-8 MΩ from borosilicate glass capillaries (Hilgenberg, Malsfeld, Germany), and filled with intracellular solution (130 mM KMeSO_4_, 10 mM HEPES, 7 mM KCl, 2 mM ATP-Na, 2 mM ATP-Mg, 0.5 mM GTP, 0.05 mM EGTA, and in some cases 10 mM biocytin). Signals were amplified using an Axoclamp 2B amplifier (Molecular devices – West Berkshire, UK) and digitized by a Micro 1401 (Cambridge Electronic Design – Cambridge, UK) at 25 kHz. Drift in membrane potential, corrected for by spike thresholds, between the start and end of recordings was 0.9 ± 1 mV, with an average duration of 14 ± 4 minutes, and access resistance of 36 ± 19 MΩ.

#### Sniff measurement

To minimally perturb sampling behavior, sniffing behavior was recorded either with a pressure sensor or flow sensor (Sensortechnics – Rugby, UK), externally located in close proximity to the left naris (contralateral to recording side). The precise orientation relative to the nostril was manually optimized prior to each recording in order to acquire the full sniff waveform in spite of any movement of the naris.

#### Double tracheotomy

Two mice were anaesthetized with ‘sleep-mix’ (0.05 mg/kg Fentanyl, 5 mg/kg Midazolam, 0.5 mg/kg Medetomidine), and both local and general analgesia applied as above for head-fixation. After the head-plate surgery, a double tracheotomy was performed by exposing the trachea and inserting two catheters, one directed to the lungs through which the mouse could freely breathe, and the other directed to the nasal passages through which flow was controlled. To mimic sniffing, a peristaltic pump (Ismatec, Wertheim, Germany) was used to generate flow inward through the nares, with a flow controller to buffer out fluctuations and the periodic opening of a 3-way valve used to simulate regular inhalations, either at 3.3 Hz (100 ms opening times), or 6.6 Hz (50 ms opening times).

#### Number of recordings

Altogether we report here recordings from 66 mitral and tufted cells. We report data from 42 cell-odor pairs from behaving animals over the timescale of learning (21 cells from 20 animals), 46 cell-odor pairs from passively exposed animals (23 cells from 20 animals), 8 cell-odor pairs from animals undergoing the task engagement/disengagement paradigm (4 cells from 4 animals), 30 cell-odor pairs from passive mice undergoing the unexpected puff experiment (23 cells from 20 animals), and 9 cells from two anaesthetized mice with a double tracheotomy. None of these cohorts are overlapping. Of the cells from mice across learning, 2 were excluded from any sniff analysis due to poor sniff signals (resulting in 38 cell-odor pairs, 20 accompanied by small (< 20 ms) sniff changes, 18 by large sniff changes), and 2 were excluded similarly from the passively exposed dataset (42 cell-odor pairs).

### Quantification and Statistical Analysis

All data was pre-processed in Spike2 version 7.1 (Cambridge Electronic Design – Cambridge, UK) and analyzed in MATLAB 2015b (MathWorks - Massachusetts, USA) and R using custom scripts and functions.

#### Statistics

In all cases, 5%–95% confidence intervals were used to determine significance unless otherwise stated. In all figures, a single asterisk denotes p < 0.05, double asterisk denoted p < 0.01 and a triple asterisk denotes p < 0.001. Where these are preceded by ‘SD’, the p value refers to the variances rather than the averages of the datasets. Means and error bars showing a single standard deviation either side are used in all cases for normally distributed data (as tested for using a Lilliefors test) of equal variance. The test for variance depended on whether the dataset was deemed normally distributed or not using the Lilliefors test – in the case of normal distributions, a Bartlett test was conducted, and in the case of non-normal distributions, a Brown-Forsythe test was conducted. Average values were then statistically compared using tests depending on whether a) the data was normally distributed and b) displayed equal variance. If both were true, unpaired two-sided Student’s t tests were used for comparison of means, unless otherwise stated. For datasets which violated at least one of these assumptions, Ranksum tests were used to compare the medians, unless otherwise stated. Where boxplots are used to represent data, the median is plotted as a line within a box formed from 25^th^ (q1) and 75^th^ (q3) percentile. Points are drawn as outliers if they are larger than q3 + 1.5 x (q3 - q1) or smaller than q1 – 1.5 x (q3 - q1). To determine points of significant difference between cumulative histograms, a bootstrapping method was used. First, data underlying the two histograms would be shuffled between datasets, and cumulative histograms would be calculated from these shuffled sets. The difference at each point between the two histograms would then be calculated. This was repeated 10,000 times, and the differences between the real cumulative histograms would then be compared to the shuffled distribution at each point. An arrow was drawn on the points at which the actual difference exceeded the 99^th^ percentile of the shuffled distribution.

#### Sniffing analysis

To extract inhalation durations, first inhalation peaks were detected as any peak above a certain threshold set according to the amplitude of the signal. Inhalation onset was set at the nearest point pre-peak that the flow trace crossed zero, while inhalation offset was set at the nearest point post-peak that the flow trace crossed zero. The distance between these points was taken as the inhalation duration. The mean inhalation duration for the first 500 ms of each odor presentation was calculated from the duration of all complete inhalations within that time period.

#### Principal cell identification

Mitral and tufted cells were distinguished from interneurons as previously (Kollo et al., 2014). The current dataset was pooled with the entire dataset of neurons recorded in the OB of awake mice acquired previously (Kollo et al., 2014), and independent component analysis was performed on the AHP waveform (2 to 25 ms from spike onset) to reveal three independent components, upon which hierarchical cluster analysis was used to band the cells into two groups, ‘principal’ and ‘other’. Based on cell morphologies from the previous dataset, and an additional 11 acquired in the current dataset, 100% of the 22 morphologies obtained from the ‘principal’ group were confirmed as mitral/tufted cells, while 86% of the 11 morphologies from the ‘other’ group were confirmed interneurons. Morphologies from the current dataset were acquired as previously (Fukunaga et al., 2012, Kollo et al., 2014): mice were perfused following recordings with cold phosphate-buffered saline, followed by 4% (wt/vol) paraformaldehyde solution in phosphate-buffered saline. Fixed OBs were embedded in porcine gelatin (10% wt/vol), before being fixed overnight in 4% paraformaldehyde. The OBs were then cut into 150 μm slices with a vibratome (Thermo Scientific – Massachusetts, USA) and stained with avidin-biotinylated peroxidase (ABC kit - Vector Labs, California, USA) and the DAB reaction. Biocytin-stained cells were traced using a Neurolucida system (MBF Bioscience, Vermont, USA). Principal cells were identified via soma size, cell body location with respect to the mitral cell layer, an apical dendrite reaching the glomerular layer and lateral dendrites branching in the external plexiform layer. MCs were distinguished from TCs based on proximity to the mitral cell layer.

#### Passive and spontaneous properties

Within the first 30 s of the recording, a current-voltage curve was calculated (Figure S4). In current-clamp, a series of current steps were applied to the cell three times, starting from −0.2 nA and increasing in 0.05 steps to 0.15 nA. At each 10 ms time point, the mean membrane potential (averaged across the three repeats) was plotted against the applied current, and a linear regression fitted. Where this model intersected the y axis (at 0 nA), the resting membrane potential was estimated. The slope of the curve represented the linear sum of input resistance and access resistance. To estimate the relative contribution of the two resistances, the mean voltage response waveform was taken for the most hyperpolarising (−0.2 nA) current pulse. A double exponential curve was fitted to the voltage trace between 0 and 20 ms, with a slow and fast time constant. The relative amplitude of the slow and fast components represented the relative contribution of input resistance and access resistance respectively, and was used to calculate each. The time constant for the slow exponential was used as a measure of membrane time constant. Spontaneous FR was calculated as the average FR in the 4 s prior to odour stimulus across all trials.

#### Odor responses and changes

For all analyses, the first presentation of each odor was excluded due to the elicitation of high frequency sniffing by the novel odorant, which rapidly decayed by the second presentation (Wesson et al., 2008). General response calculations: All traces were aligned to first inhalation onset following final valve opening. For V_m_ response calculations, spike waveforms, including the AHP, were subtracted from the V_m_ trace (−5 to 20 ms after spike peak; Figures S3D and S3E). Responses for each trial were calculated as the mean V_m_ within the first 500 ms post odor onset, normalized to the baseline membrane potential in the 2 s prior to odor onset. FR responses were calculated as the mean number of spikes per 0.25 s time bin in the first 500 ms post odor onset, normalized to that calculated for 2 s prior to odor onset. Significant responses were determined for both V_m_ and FR using a paired t test to compare baseline and odor-evoked activity for all trials. For response changes across learning: Significant changes between early and late trials for each odor response were identified by comparing the five ‘early trials’ in block 1 (stimulus presentation #2 to 6), with the 5 last presentations of the stimulus (‘late trials’). Significant change was determined using an unpaired t test, p < 0.05. To determine onset of response change: For each response, the mean V_m_ response waveform calculated for early trials was subtracted from that calculated from late trials, to generate a response change waveform at each time-point from odor onset. This was then normalized by the standard deviation of this resulting waveform during the baseline period 2 s prior to odor onset. Response change onset was detected where the response change magnitude first exceeded 2 standard deviations and remained there for at least 50 ms. For task engagement/disengagement changes: The first 500 ms of the stimulus was analyzed for V_m_ responses, and the full 2 s for FR responses. 5 trials of initial engagement were defined as the last 5 trials of each stimulus prior to physical port removal, disengagement trials were defined as 5 trials with at least 3 consecutive misses within the block, and re-engagement trials were based on the first 5 trials of the stimulus after the mouse initiates licking after port return.

#### Detectability and discriminability analysis

Visual aid for this analysis is in Figure S13. For each response, five mean V_m_ response waveforms were generated from different sets of 3 early trials, 3 mid-point trials and 3 late trials (aligned to first inhalation post odor onset). Five mean baseline waveforms were similarly generated from V_m_ traces (triggered by a random inhalation onset) during the ITIs corresponding to the trials from which odor response waveforms were drawn. Population response vectors were then constructed from these mean response waveforms for all cell-odor pairs recorded. At each time point relative to inhalation onset, the Euclidean distance was calculated between response and baseline vectors, and this was repeated for each trial subset to gain a mean detectability over time, and a standard deviation. The average baseline Euclidean distance 200 ms prior to odor onset was subtracted from the trace, normalizing the baseline to zero. Peaks of detectability were defined as the maximum detectability within the first 170 ms after odor onset. Discriminability was analyzed similarly, however the response vectors used to calculate the Euclidean distances were calculated between CS+ and CS- mean V_m_ response waveforms for the five sets of early, mid-point and late trials, i.e., the Euclidean distance was generated between population responses for CS+ and CS- separately. Where we analyzed changes in discriminability for cell-odor pairs recorded alongside large and small changes in MID (Figure 4H), only cases where ΔMID was large for both odors were considered in the large ΔMID group. This is because cases showing a large ΔMID only for one odor represent selective sniffing responses to the CS+, i.e., occurring after the first sniff and after discrimination has already been made – thus changes of this nature would not give rise to changes in representation in the timescale analyzed.

For detectability and discriminability of FR responses, FR responses were calculated by first counting the number of spikes in 50 ms time bins, and then averaging these spike counts across 5 sets of trials from early and late time periods. A moving average was then applied to these averaged spike counts with a 200 ms (4 time bin) window. Next, the resulting averages were converted to Z-scores by taking each average spike count waveform for a given cell odor pair and subtracting the mean of all time bins and dividing by the standard deviation of all time bins. Euclidean distances were then calculated from the population vectors as for membrane potential data. Euclidean distances were normalized to baseline by subtracting the average Euclidean distance in the 1 s prior to odor onset, and peak values detected within the first 250 ms from odor onset.

#### Sniff-V_m_ modulation amplitudes and preferences

Visual aid for this analysis is in Figure S11B. The sniff-V_m_ modulation properties of each cell were calculated as previously (Fukunaga et al., 2012). Baseline sniff-V_m_ modulation: due to the high variability of sniff behavior in awake mice, analysis was restricted to sniff cycles between 0.25 and 0.3 s in duration, where also the preceding sniff cycle was within this range. Mean V_m_ from the spike-subtracted V_m_ trace was taken as a function of sniff cycle phase for at least 150 sniffs, and this was plotted as Cartesian coordinates. The angle of the mean vector calculated by averaging these Cartesian coordinates was taken as the phase preference of the cell, while the difference between the mean V_m_ at the preferred phase, and the minimum value on the mean V_m_ waveform was taken as the amplitude of modulation. Odor sniff-V_m_ modulation: This was calculated as for baseline, but based on the first four sniffs post odor onset for the 10 trials of lowest sniff rates. As odor responses can have both tonic and sniff-modulated components, the phase-V_m_ trace for each sniff had to be normalized according to the linear vector connecting the V_m_ at the beginning and end of the sniff. To determine significance, a bootstrapping method was used: 100 ms segments of V_m_ data were randomly selected for each cell and connected to form a shuffled dataset. The phase analysis was then performed on these shuffled datasets, and a modulation amplitude calculated and this was repeated 100 times. Significant modulation was assigned when the actual modulation amplitude exceeded that of the 95^th^ percentile of shuffled data amplitudes.

#### Putative mitral cell versus tufted cell identification

For each ITI, the mean V_m_ was calculated during sniffs of duration of < 200 ms where also the preceding sniff was within this duration range (‘fast sniffs’). This mean V_m_ was then normalized by subtracting the mean V_m_ during sniffs of duration 0.25 and 0.3 s within the same ITI to calculate the ‘fast-sniff evoked V_m_’. Only cells with at least 20 such ‘fast sniffs’ within the recording were considered for the analysis. To determine significance, a bootstrapping method was used: the mean V_m_ for all sniffs within a trial was randomly shuffled, and the shuffled data analyzed as before 100 times. The actual fast-sniff evoked V_m_ was then compared to the 5^th^ and 95^th^ percentiles of the shuffled distribution in order to assign significance.

We noted that, consistent with anaesthetized mice (Fukunaga et al., 2012), there was a bimodal distribution of phase preferences for the sniff cycle in baseline membrane potential, one within exhalation phase, and another within inhalation phase. We hypothesized that these may correspond to MC and TC phenotypes respectively, as reported previously for anaesthetized animals (Fukunaga et al., 2012). The phase boundaries for separating putative MCs and TCs were selected as those which produced the lowest p value when comparing the V_m_ change during fast sniffing (in absence of odor) for cells in each of the two boundaries (rank-sum test). The putative assignment to MC or TC was confirmed morphologically for 8 cells (Figure 7F), with MC and TC distinction based largely on soma location relative to the mitral cell layer, as dendritic reconstruction was in many cases incomplete.

#### Unexpected tactile stimulus experiments in passive mice

In 10 passive mice, odors were presented as before, but this time with a random chance of an unexpected tactile stimulus to accompany the odor (25% chance) to evoke fast sniffing. Since the sniffing response to the tactile stimulus eventually habituated, for each response, the five trials with lowest MID were selected and compared to the five trials with highest MID. The difference in response for these sets of trials was then calculated for the first 500 ms of the stimulus as for learning mice.

#### Reaction times

Reaction time calculations were based on 10 or more trials of 80% correct performance. From lick behavior: For each CS+ and CS-, lick probability was calculated in a moving time window of 100 ms, aligned to the first inhalation after final valve opening. The difference between the probability of licking for CS+ and CS- for each time window was calculated, and the leading edge of the first window at which this calculated difference significantly deviated from the values calculated from the 2 s window prior to odor onset was considered the reaction time (Figure S2A). From sniff behavior: Inhalation and exhalation duration values were calculated for CS+ and CS- as a function of sniff number from odor onset. These values were compared between those calculated for CS+ and CS- using a t test, and the decision time was calculated based on the first inhalation or exhalation within the series to show a significant difference (Figure S2B). For 12/21 mice there was a significant difference between CS+ and CS- sniffing within the first two sniff cycles.

## Supplemental Information

Document S1. Figures S1–S13

Document S2. Article plus Supplemental Information
