# Supplementary material for: Active Sampling State Dynamically Enhances Olfactory Bulb Odor Representation
Source: Neuron. 2018 Jun 27;98(6):1214–1228.e5. doi: 10.1016/j.neuron.2018.05.016 (PMC6030445; doi:10.1016/j.neuron.2018.05.016)
Supplement: Document S1. Figures S1–S13 [file mmc1.pdf]

Neuron, Volume *98*

Supplemental Information

Active Sampling State Dynamically

Enhances Olfactory Bulb Odor Representation

Rebecca Jordan, Izumi Fukunaga, Mihaly Kollo, and Andreas T. Schaefer

Supplementary figures

A

	Odor	Vapor pressure (mmHg)	% vol
Mixture 1	methyl salicylate	0.034	15
	eugenol	0.022	23
	cinnamaldehyde	0.029	18
	creosol	0.024	22
	1-nonanol	0.023	22
Mixture 2	1-heptanol	0.22	25
	0-ethylphenol	0.15	35
	acetophenone	0.37	14
	ethyl heptanoate	0.2	26
Mixture 3	guaiacol	0.11	24
	valeric acid	0.2	14
	(+)-carvone	0.16	17
	2-phenyl ethanol	0.09	32
	4-allyanisole	0.21	13
Mixture 4	α-terpinene	1.66	20
	butyric acid	1.65	19
	limonene	1.55	21
	benzaldehyde	1.5	21
	eucalyptol	1.65	19

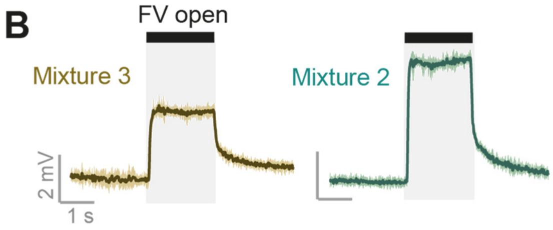

Figure S1. Odor stimuli, related to STAR methods.

(**A**) Each table shows the components added to each of the four odor mixture stimuli. The components were added in inverse proportion to their relative vapor pressure, such that higher vapor pressure components were added in lesser volume. This was done in attempt to keep the relative contribution of each component fairly similar in the olfactory headspace. (**B**) Example average mini-photoionisation detector signals in response to 15 odor presentations of mixture 3 (left) and mixture 2 (right). Solid line indicates mean signal, shaded areas represent standard deviation. 'FV open' and shaded region indicates where the final valve was opened. The magnitude of each square pulse was calibrated to 1% of the maximum signal recorded when exposed to saturated vapor pressure.

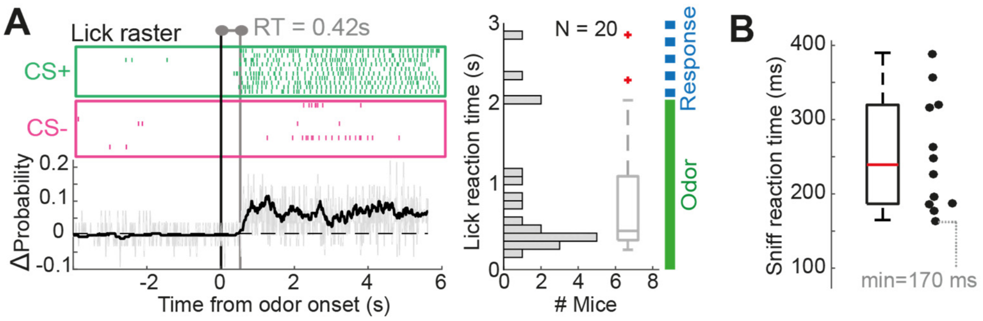

Figure S2. Reaction times in go/no-go task, related to STAR methods.

(**A**) Left: example reaction time (RT) calculation for one mouse. Lick rasters for CS+ and CS- during criterion performance are compared. Bottom trace: grey: shows for each 10 ms time window the difference in probability of lick occurrence between CS+ and CS-. In black: moving average of the grey trace with a sliding time window of 100 ms. Reaction time is calculated where this exceeds 2x the SD of baseline. Right: distribution of reaction times calculated for 20 mice. (**B**) Reaction times calculated from the time of first detectable significant deviation between CS+ and CS- sniffing traces (which usually occurred within the first exhalation period) for 12 mice (see STAR methods).

.

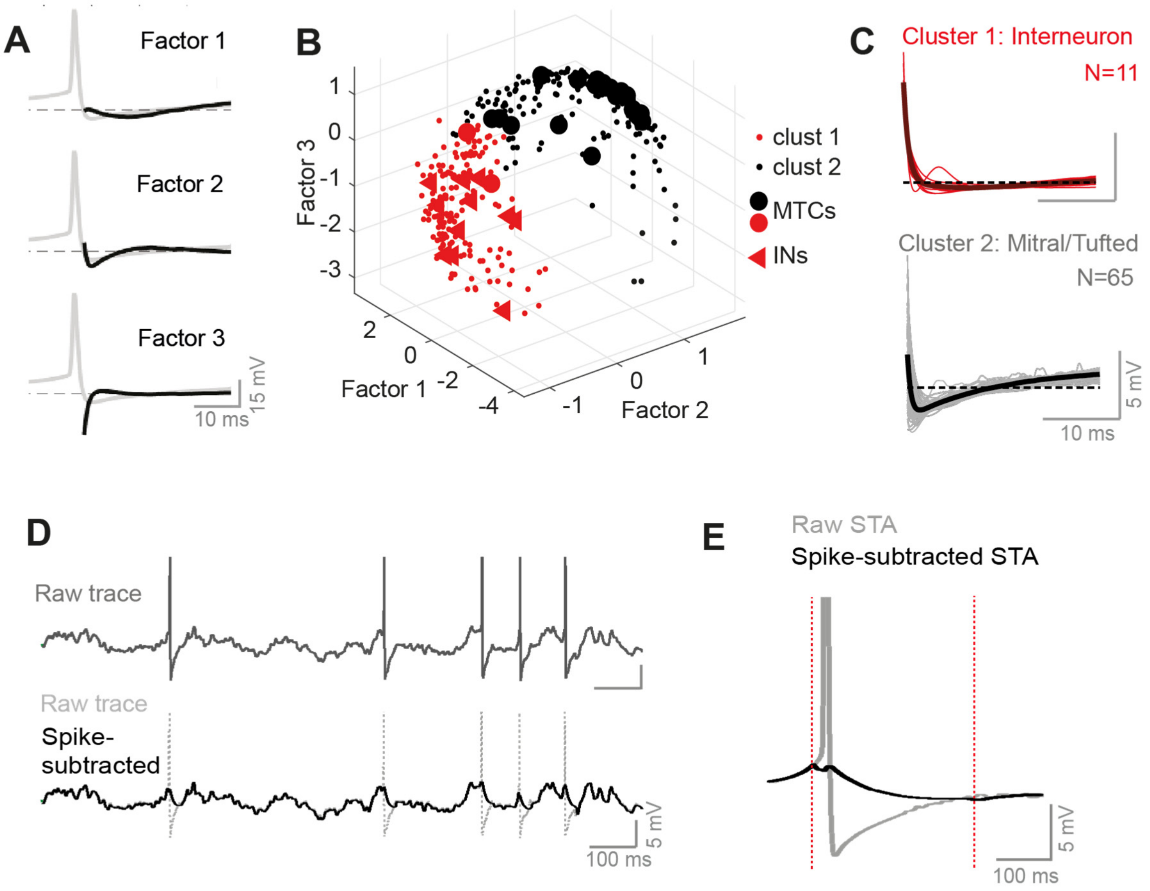

Figure S3. Principal cell (mitral/tufted cell) identification and spike subtraction, related to STAR methods.

Data from the current study (N = 76 cells; 66 cells reported in the main analyses within the study, as well as an additional 10 cells not reported elsewhere) was pooled with electrophysiological data from Kollo et al, 2014 (N = 325 cells) for the purpose of cell type identification (see STAR methods). (**A**) Three factors identified using independent component analysis on AHP waveforms (2 to 250 ms from onset of action potential) from the entire dataset. (**B**) Scatter of the weights of each factor for all cells in the dataset (N = 401). Hierarchical cluster analysis was used to divide the dataset into two clusters based on these weights. In red is cluster 1, and described interneuron-like AHPs (IN); in grey is cluster 2, describing MTC-like AHPs. Larger points show data for recovered morphologies from both the previous dataset (n = 22; Kollo et al., 2014) and newly recovered morphologies (n = 11). Circles show identified MTCs, and triangles show identified interneurons. All 20 cell morphologies (9 from the data in the main manuscript, 11 from Kollo et al. 2014) recovered from cells in the MTC cluster were confirmed MTCs – thus identification of principal cells occurred with a 0% false positive rate. However, identification of INs occurred with an 18% false positive rate (2/11 morphologies recovered from the IN group were MTCs). This indicates a high threshold for inclusion into the MTC group, and more errors of omission than errors of inclusion. (**C**) AHPs from the current dataset that fall into either cluster 1 (red, INs), or cluster 2 (grey, mitral and tufted cells). Thick black plot shows the average of all AHPs in the cluster. (**D**) Top: example raw membrane potential trace prior to spike subtraction. Below: black trace shows membrane potential after spike subtraction. Raw trace is shown in dotted grey for comparison. Spikes are clipped for display (**E**) Spike-triggered average (STA) of membrane potential traces both prior to spike subtraction (grey) and after spike subtraction (black), corresponding to example cell in D.

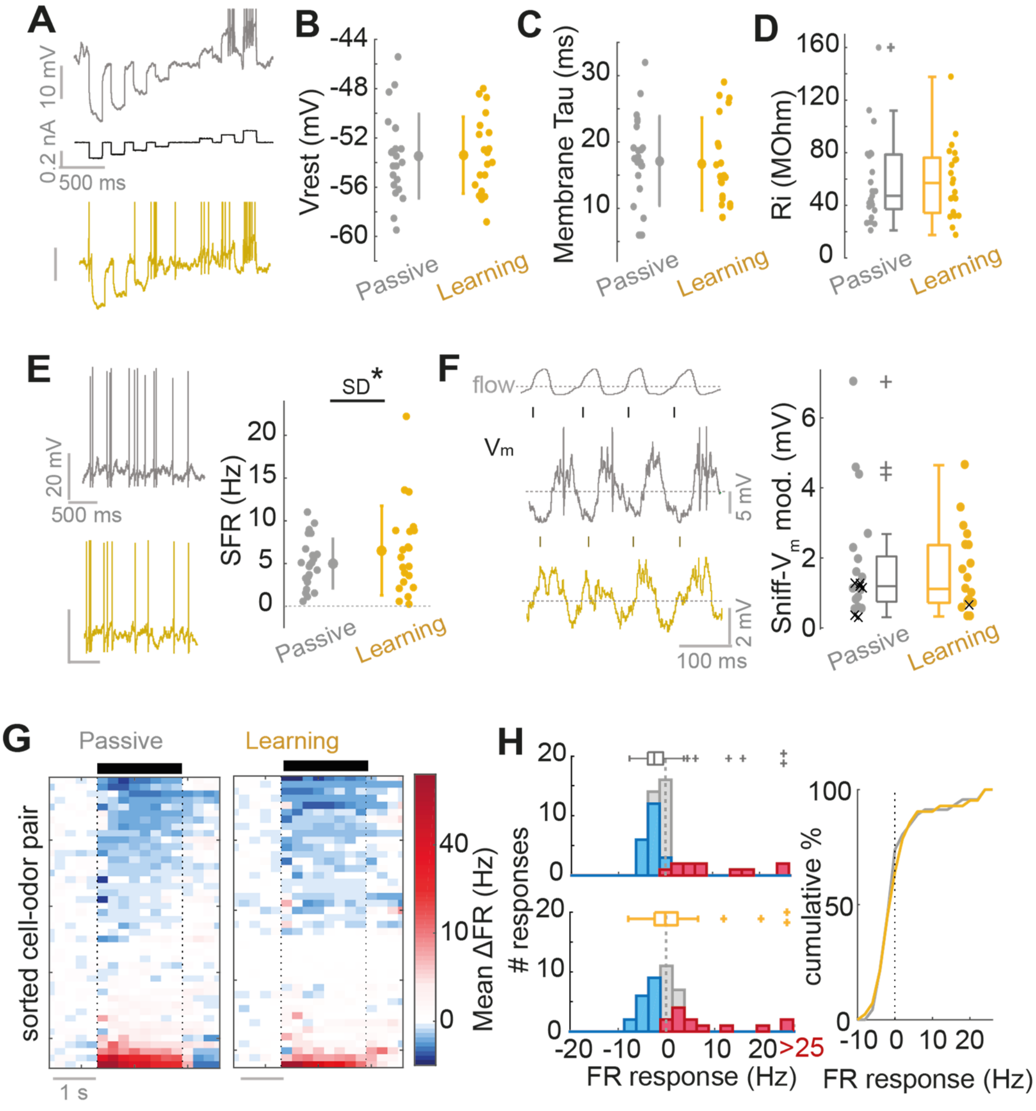

Figure S4. Basic properties of MTCs do not overtly differ between passive and learning states, related to Figure 1.

(**A**) Example current steps and concurrent voltage responses measured in current clamp for example MTCs recorded in a passive mouse (grey) and a learning mouse (gold). Spikes have been clipped for display. (**B**) Resting membrane potential (V_{rest}) compared for passive (N = 23) and learning MTCs (N = 21). p = 0.94, unpaired t-test; p = 0.68, Bartlett test. (**C**) Comparison of membrane time constants (Tau). p = 0.81, unpaired t-test; p = 0.86, Bartlett test. (**D**) Comparison of input resistance (R_i). p = 0.68, Ranksum; p = 0.54, Brown-Forsythe test. (**E**) Left: example baseline firing activity from two MTCs. Right: comparison of spontaneous firing rates (SFR) across the two datasets. p = 0.31, unpaired t-test; p = 0.01, Bartlett test. (**F**) Left: example V_m traces during the inter-trial interval across 4 sniff cycles for 2 MTCs, one recorded in a passive mouse (grey), the other during learning (gold). Flow trace shows nasal flow, black ticks show inhalation onsets. Spikes have been clipped for display purposes. Right: sniff-V_m modulation amplitude compared for passive and learning mice. Crosses show points which did not show significant modulation. p = 0.9, Ranksum; p = 0.8, Brown-Forsythe test. (**G**) Heatmap of FR responses averaged across all trials for each cell-odor pair, sorted by mean 2 s FR response, for both passive (n = 46) and learning (n = 42) datasets. Black bar indicates odor stimulus. (**H**) Left: histograms of average 2 s FR responses (average FR during the 2 s stimulus minus average FR 2 s prior to stimulus) for (top) passively exposed and (bottom) learning mice. Right: cumulative histograms comparing average FR response data for passive (grey) and learning (gold) cell-odor pairs.

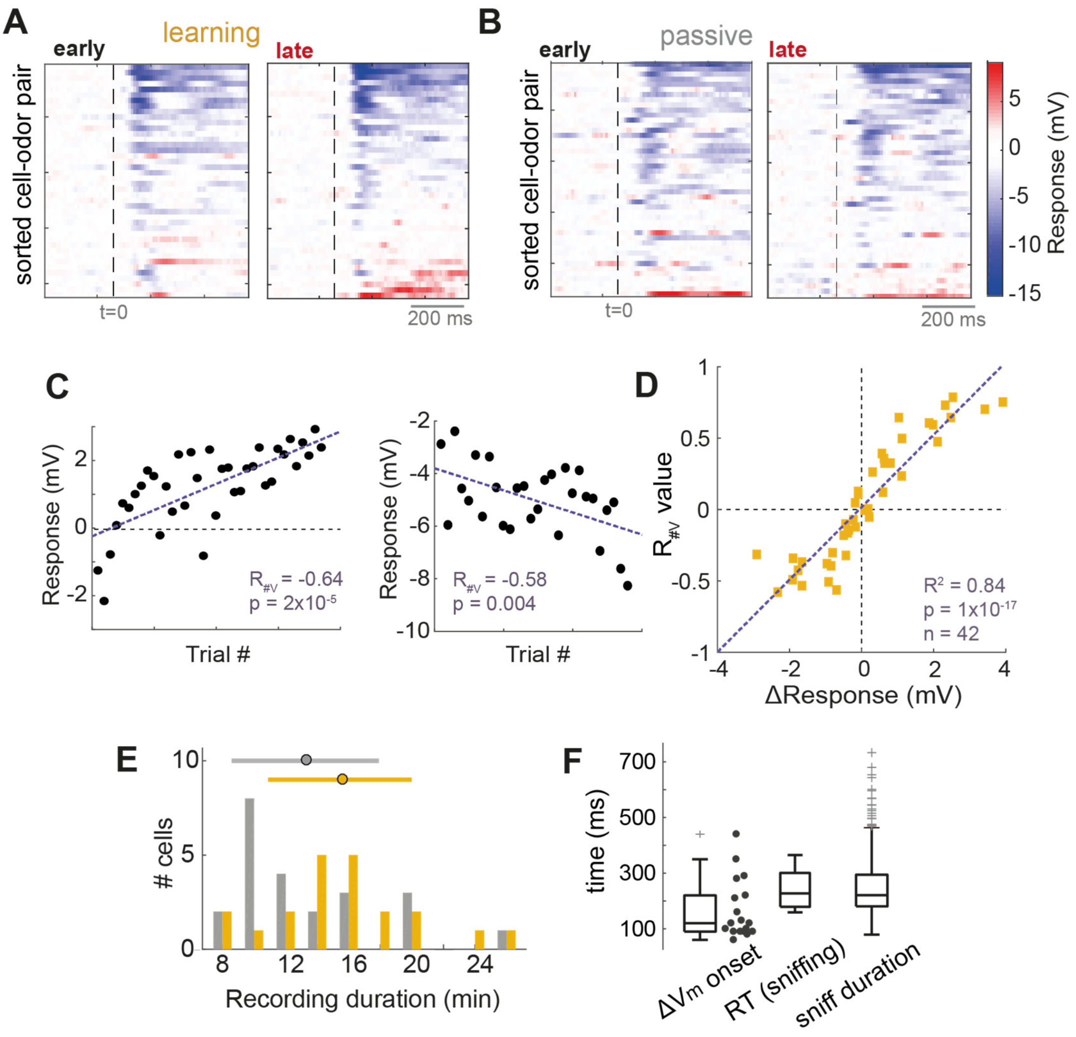

Figure S5. Additional data for learning response changes, related to Figure 2.

(**A**) Heat-map of average V_m responses across whole dataset for both early (left) and late (right) trials in learning mice, sorted by their mean late response. Dashed lines indicate start of the odor stimulus (t = 0; aligned to first inhalation onset). (**B**) As for panel A, but for cell odor pairs recorded in passively exposed mice. (**C**) Scatterplots between V_m response and trial number for two different cell-odor pairs. (**D**) Scatterplot between the response change (late - early) and the R value of the correlation between trial number and response ($R_{\#V}$) as in panel C for all cell-odor pairs in learning mice. (**E**) Histogram of recording durations for learning (gold bars) and passive datasets (grey bars). Error bars above show mean and SD for each dataset. (**F**) Comparison of response change onsets (ΔV_m onset) as shown in Figure 2G, reaction times (RT) as calculated from sniff divergence (as in Figure S2B), and sniff durations taken from the first sniff during stimulus sampling for 5 early and 5 late trials for each cell odor pair (altogether, n = 390).

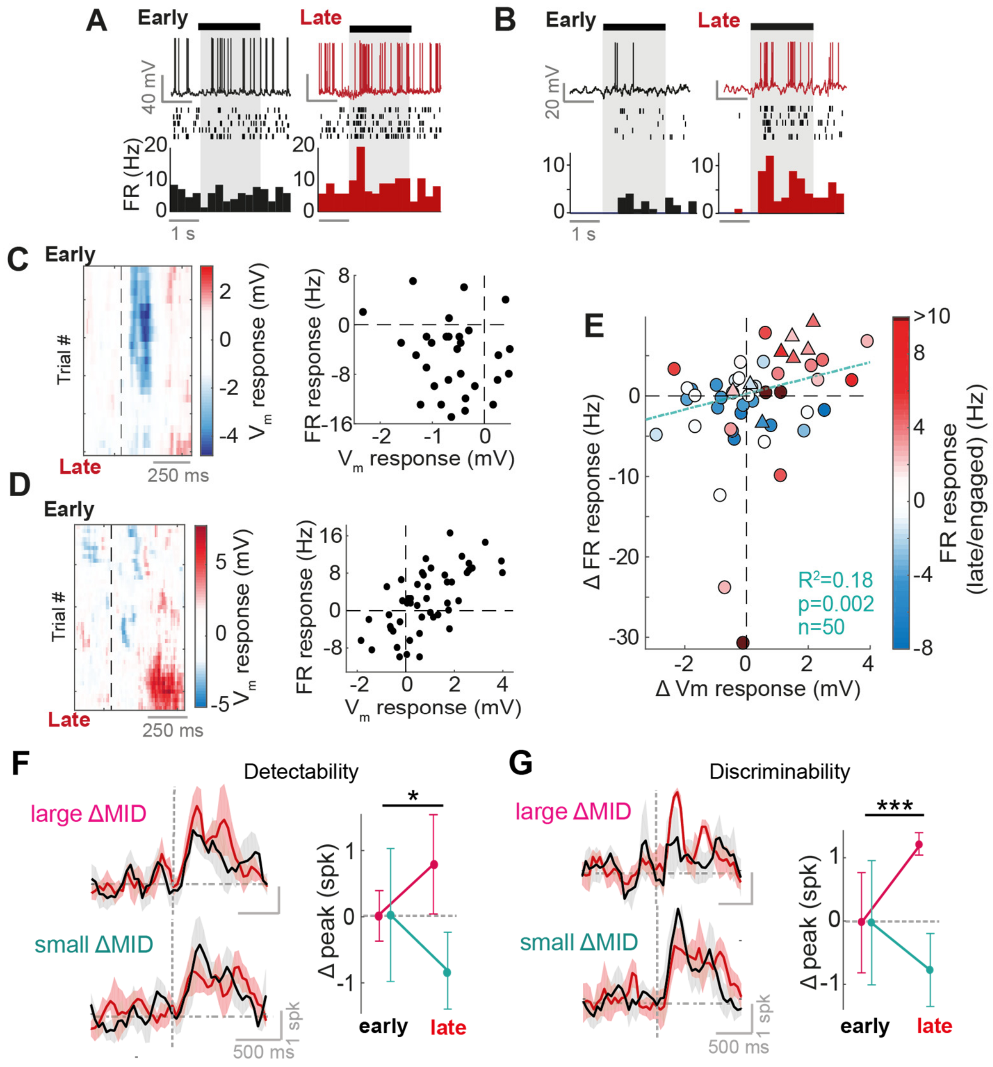

Figure S6. FR changes correspond to V_m changes across learning and result in improved odor representation, related to Figure 2 and 4.

(**A**) Example cell-odor pair displaying a significant increase in excitatory firing rate response between early and late trials. Top shows an example V_m trace for one trial, and below shows a raster of spikes across five trials and corresponding peristimulus time histogram. (**B**) As for panel A, but for a different cell-odor pair. (**C**) Left: heatmap depicting 5-trial moving average of V_m response for an example cell-odor pair undergoing significant reduction in inhibition. Right: scatterplot between mean 500 ms V_m response and 500 ms FR response for the same cell-odor pair across trials. (**D**) As for panel C, but for a cell undergoing significant increase in excitation. (**E**) Scatterplot between the FR response change, and the V_m response change across learning (late - early) (circles, n = 42) and between task engaged and disengaged trials (triangles, n = 8). Points have been colored according to their FR response in late or engaged trials (blue = inhibitory responses, red = excitatory responses). (**F**) (Corresponds to Figure 4G) Left: Euclidean distance between population response vectors and baseline data (measure of response detectability), split into cell-odor pairs recorded alongside large MID change (>20 ms change, n = 18), and small MID change (<20 ms change, n = 20) for early (black) and late (red) trials Shading shows SD. Right: plot to show change in peak detectability within the first 170 ms of the stimulus across early and late trials (relative to mean for early trials). Error bars show SD. Large ΔMID: early = 2.1 ± 0.4 spk, late = 2.9 ± 0.8, Cohen's d = 1.1, n = 5; small ΔMID: early = 2.6 ± 1.0 spk, late = 1.7 ± 0.6 spk , Cohen's d = -1.0; significant interaction (ΔMID x time): p = 0.02, df = 1, F = 6.6, two-way ANOVA. *Legend continues on next page.*

(**G**) (Corresponds to Figure 4H) As for panel F, but for the Euclidean distance between population response vectors for CS+ and CS- (measure of response discriminability) for data that is split into cell-odor pairs recorded alongside large MID change (>20 ms change for both CS+ and CS-, n = 8 cells) and small MID change (any other cell, n = 11 cells). Large ΔMID: early = 1.6 ± 0.8 spk, late = 2.8 ± 0.2; Cohen's d = 1.6, n = 5; small ΔMID: early = 3.1 ± 1.0 spk, late = 2.3 ± 0.6 spk, Cohen's d = -1.6; significant interaction (ΔMID x time): $p = 8 \times 10^{-6}$, df = 1, F = 41, two-way ANOVA.

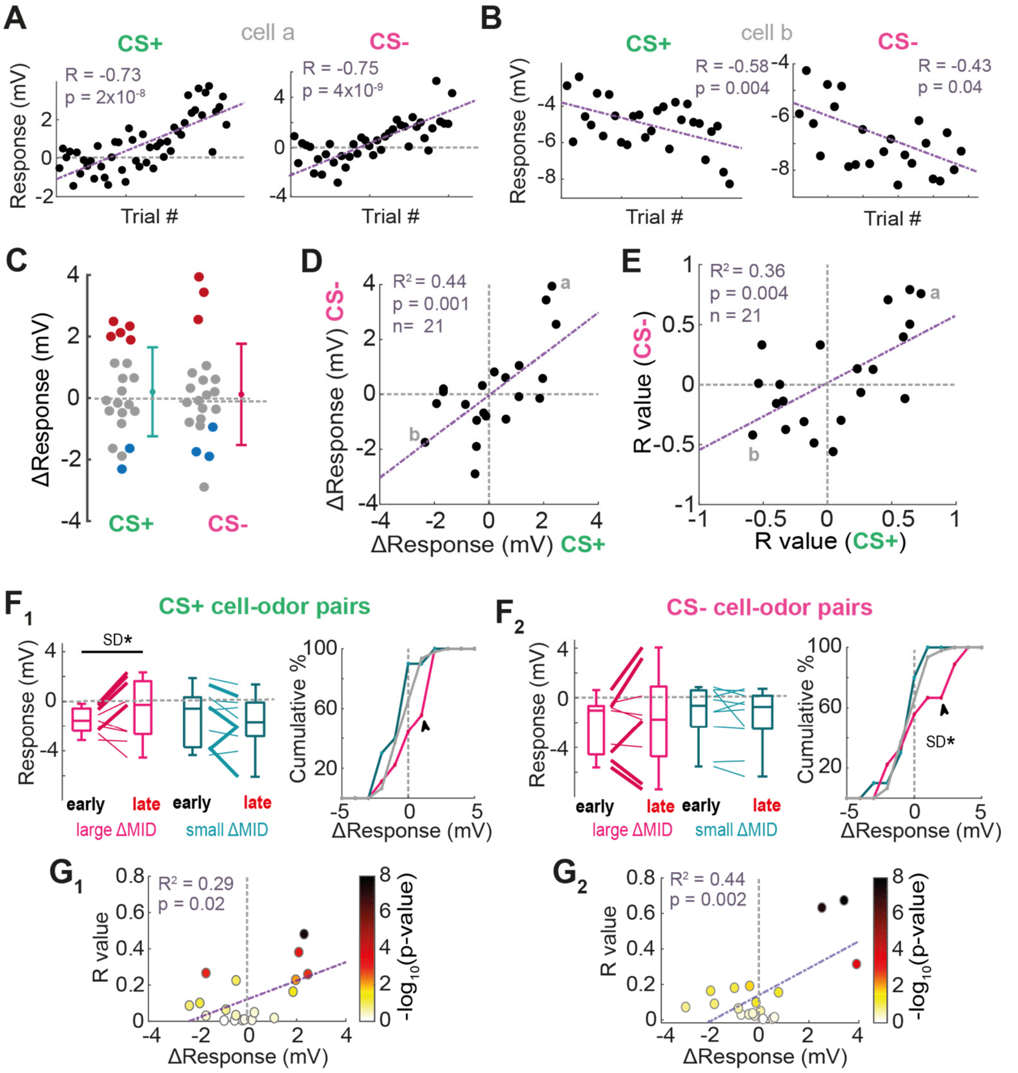

Figure S7. Response changes do not depend on reward contingency, related to Figure 2 and 4.

All data comes from learning dataset. (**A**) Example scatterplots of V_m response as a function of trial number for one cell, for both CS+ odor (left) and CS- odor (right). (**B**) As for panel A, but for another cell. (**C**) Comparison of response changes (late - early) for CS+ and CS- cell-odor pairs.(**D**) Scatterplot of response change for CS+ versus response change for CS- for each cell. ‘a’ and ‘b’ indicate data points corresponding to examples in panel A and B. (**E**) Scatterplot of R values (as for regressions in panel A and B) for CS+ versus CS- odor for each cell. ‘a’ and ‘b’ indicate data points corresponding to examples in panel A and B. (**F_1**) (note: F and G correspond to results in Figure 4 of the manuscript). Left: plot of mean V_m response for CS+ cell-odor pairs for early and late trials, for those cells undergoing large ΔMID (> 20 ms) and those undergoing small ΔMID (< 20 ms). Large ΔMID: early V_m response = -1.5 ± 1.0 mV, late = -0.8 ± 2.4 mV; p = 0.02, Bartlett test, n = 9; small ΔMID: early = -1.2 ± 2.2 mV, late = -1.7 ± 2.2 mV; p = 0.96, Bartlett test, n = 10. Right: cumulative histograms to show response change (late - early) for CS+ cell-odor pairs undergoing large ΔMID (magenta) small ΔMID (cyan), and for all cell-odor pairs in passive mice (grey). Arrow indicates points of significant difference (p < 0.01, bootstrapping see STAR methods). (**F_2**) As for F_1, but for CS- data. Large ΔMID: response change = 0.6 ± 2.2 mV; small ΔMID: response change = -0.3 ± 1.0 mV; p = 0.04, Bartlett test. (**G_1**) Scatter plot between the R^2 value of the correlation between MID and V_m response across trials for each CS+ cell-odor pair, and the change in response between early and late trials. (**G_2**) As for G_1, but for CS- cell-odor pairs.

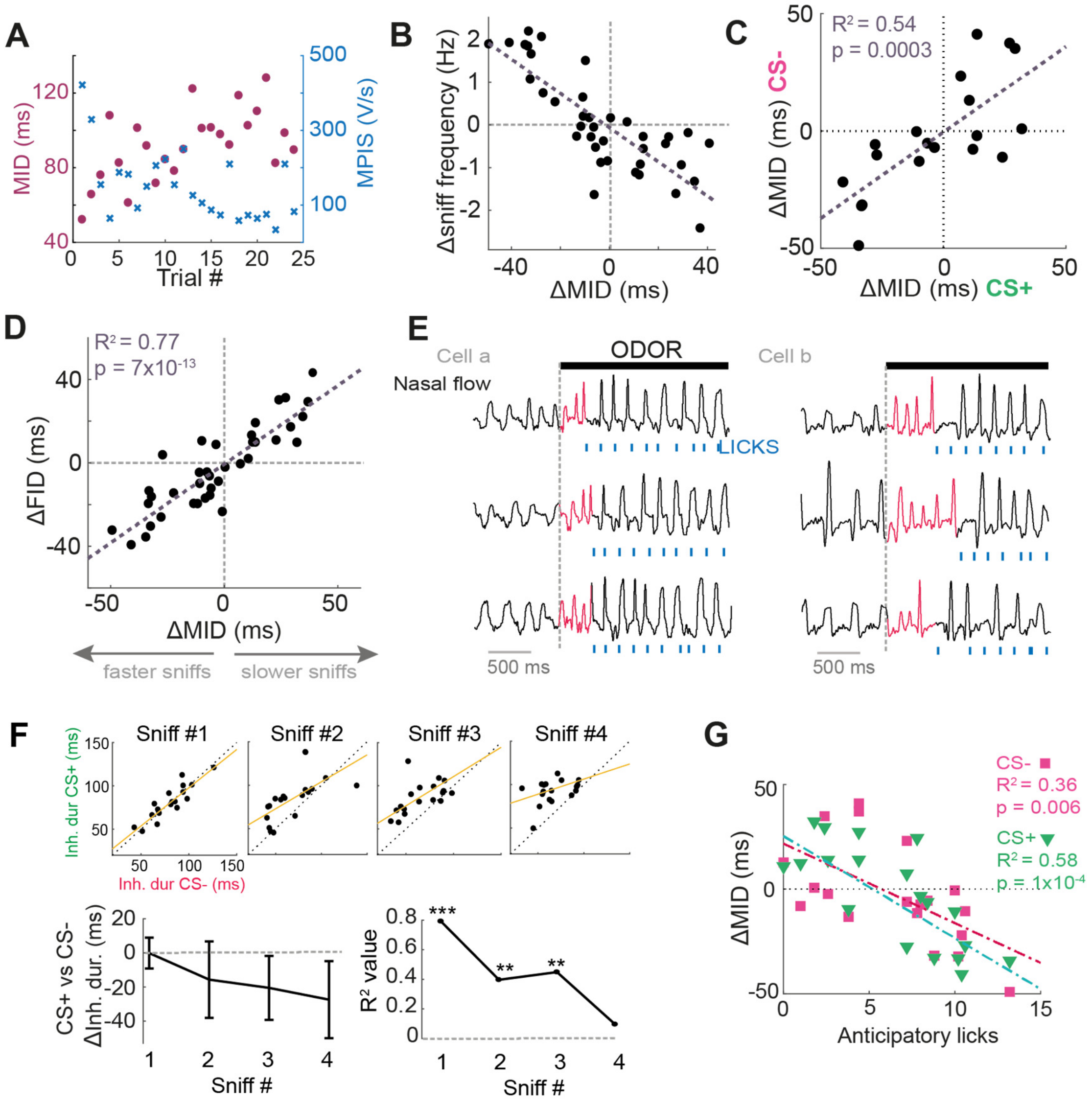

Figure S8. Additional sniffing analysis, related to Figure 3.

All data comes from learning dataset. (**A**) Left: scatterplot of MID during the odor stimulus (purple dots) and mean peak inhalation slope (MPIS; blue crosses) as a function of trial number for an example cell-odor pair. (**B**) Scatterplot showing change in MID against change in sniff frequency between early and late trials for each cell-odor pair. $R^2 = 0.59$, $p = 3x10^{-8}$. (**C**) Scatterplot between MID change for CS+ and MID change for CS- odors for each cell. (**D**) Scatterplot of changes in first inhalation duration (FID) versus changes in MID between early and late trials. (**E**) Nasal flow traces for CS+ stimuli for two example mice during criterion performance. Highlighted in red are sniffs that occur before the onset of licking (licks shown as blue dashes below each flow trace). (**F**) Scatterplots to show correlation between inhalation duration for CS+ and CS- stimuli for 18 cells, for the first, second, third and fourth sniff cycle after odor onset (from left to right). Black dotted line indicates unity, while yellow solid line indicates the linear regression for the dataset. Below left shows the average difference (across cells) in inhalation duration between CS+ and CS- stimuli as a function of sniff cycle number. Inhalation duration gradually gets larger for the CS- across the sniffs. Below right shows a plot of the R^2 value for the correlation between inhalation duration for CS+ and CS- stimuli across cells as a function of sniff number. Asterisks indicate the significance level of the correlation. (**G**) MID change across learning as a function of the mean number of anticipatory licks in late trials for CS+ trials. Green triangles shows MID data for CS+ only, and pink squares shows MID data for CS- only. Note that anticipatory licks for the CS- data were not calculated from CS- trials, but adjacent CS+ trials.

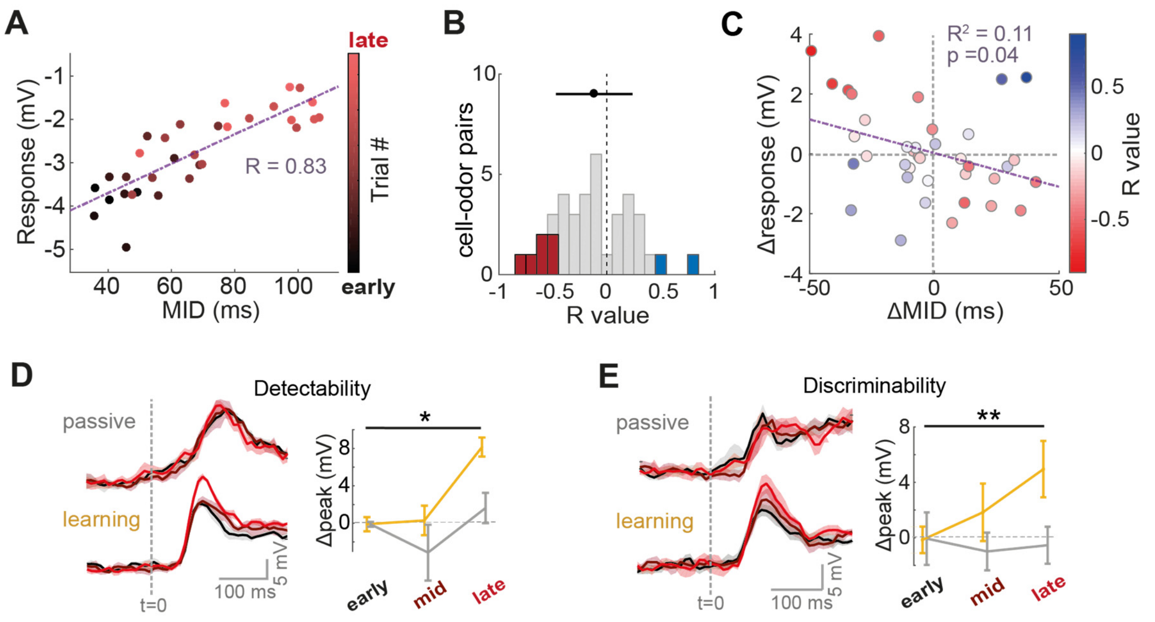

Figure S9. Additional data for relationship between ΔMID and V_m response, related to Figure 4.

All data comes from learning dataset. (**A**) Example cell-odor pair showing less common positive trend between MID and V_m response across trials. (**B**) Histogram to show R values between MID and V_m response (as in shown in panel A) for all 38 cell-odor pairs. Note that most R values are negative (V_m becoming more positive as MID is reduced). (**C**) Scatterplot between the changes in MID between early and late trials, and the corresponding changes in V_m response for each cell-odor pair. Points are colored according to the R value of the correlation between MID and V_m response across trials (as shown in panel A). (**D**) Left plots: Euclidean distance between population vectors for odor response and baseline data (see Figure S13) as a function of time since odor onset (t = 0). This gives an indication of response detectability. Black plot shows data from early trials, maroon shows data from mid-point trials, and red shows data from late trials. Right: plot to show change in peak detectability within the first 170 ms of the stimulus across early, mid-point and late trials, relative to mean for early trials. Error bars show SD. Gold plot is for learning mice (n = 42 cell-odor pairs), and grey plot is for passive mice (n = 46 cell-odor pairs). Learning: peak early = 31.9 ± 0.8 mV, late = 40.1 ± 1.0 mV, Cohen's d = 1.6, n =5; passive: peak early = 35.3 ± 0.3 mV, late = 37.1 ± 1.6 mV; Cohen's d = 1.1, n = 5; significant interaction (behavioral state x time): p = 0.02, df = 1, F = 6.3; two-way ANOVA). (**E**) As for D, but with the Euclidean distance measured between population vectors for the CS+ and CS- to give an indication of response discriminability. Learning: peak early = 19.8 ± 1 mV, late = 25.0 ± 2 mV, Cohen's d = 1.7, n = 5; passive: peak early = 20.4 ± 1.9 mV, late = 19.9 ± 1.3 mV, Cohen's d = 0.05, n = 5; significant interaction (behavioral state x time): p = 0.003, df = 1, F = 11.9, two-way ANOVA. Note that both discriminability and detectability peak at 107 ± 5 ms and 114 ± 10 ms from odor onset respectively, so this enhanced representation occurs within the timescale of decision making.

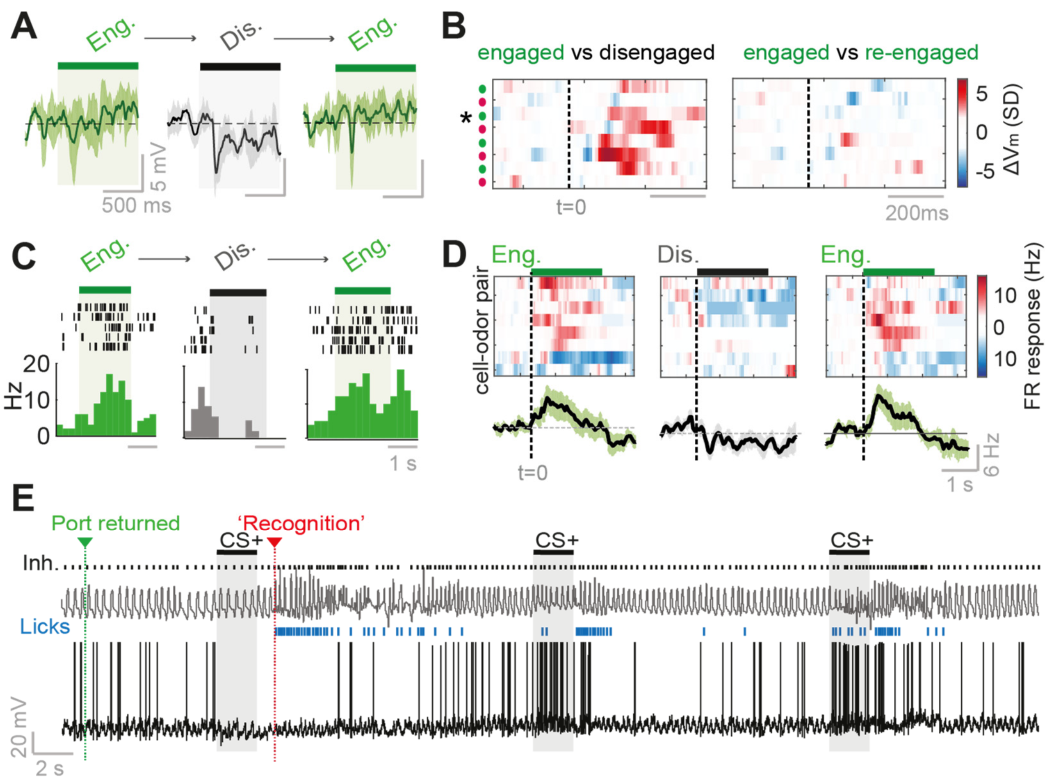

Figure S10. Additional data for task engagement/disengagement paradigm, related to Figure 5.

(**A**) Average V_m responses of an example cell-odor pair for engaged, disengaged and re-engaged trials. Shaded region shows SD. Thick bars indicate odor stimulus. (**B**) Heatmaps to show change in average V_m responses between engaged and disengaged trials (engaged – disengaged waveform), and engaged and re-engaged trials (engaged – re-engaged). Each has been normalised by the SD of the baseline changes 2 s prior to odor stimulus. Green dots on the left indicate CS+ cell-odor pairs, and pink dots indicate CS- cell-odor pairs. Asterisk indicates cell-odor pair shown in panel A. (**C**) Example cell-odor pair showing change in FR between engaged and disengaged trials. Dashes show spike rasters for five trials, and corresponding peri-stimulus time histograms are below. (**D**) Heatmaps to show FR responses for all 8 cell-odor pairs during engaged trials, disengaged trials and re-engaged trials (from left to right). Below shows average FR response across all 8 cell-odor pairs, shaded area shows standard error. (**E**) Example trace showing 3 consecutive CS+ trials across task re-engagement for one response. Port returned indicates when the reward port was returned and the task re-engaged. 'Recognition' indicates the point at which a free reward was used to indicate to the animal that the port had returned. Note the rapid switch in sign of the response after recognition of task re-engagement.

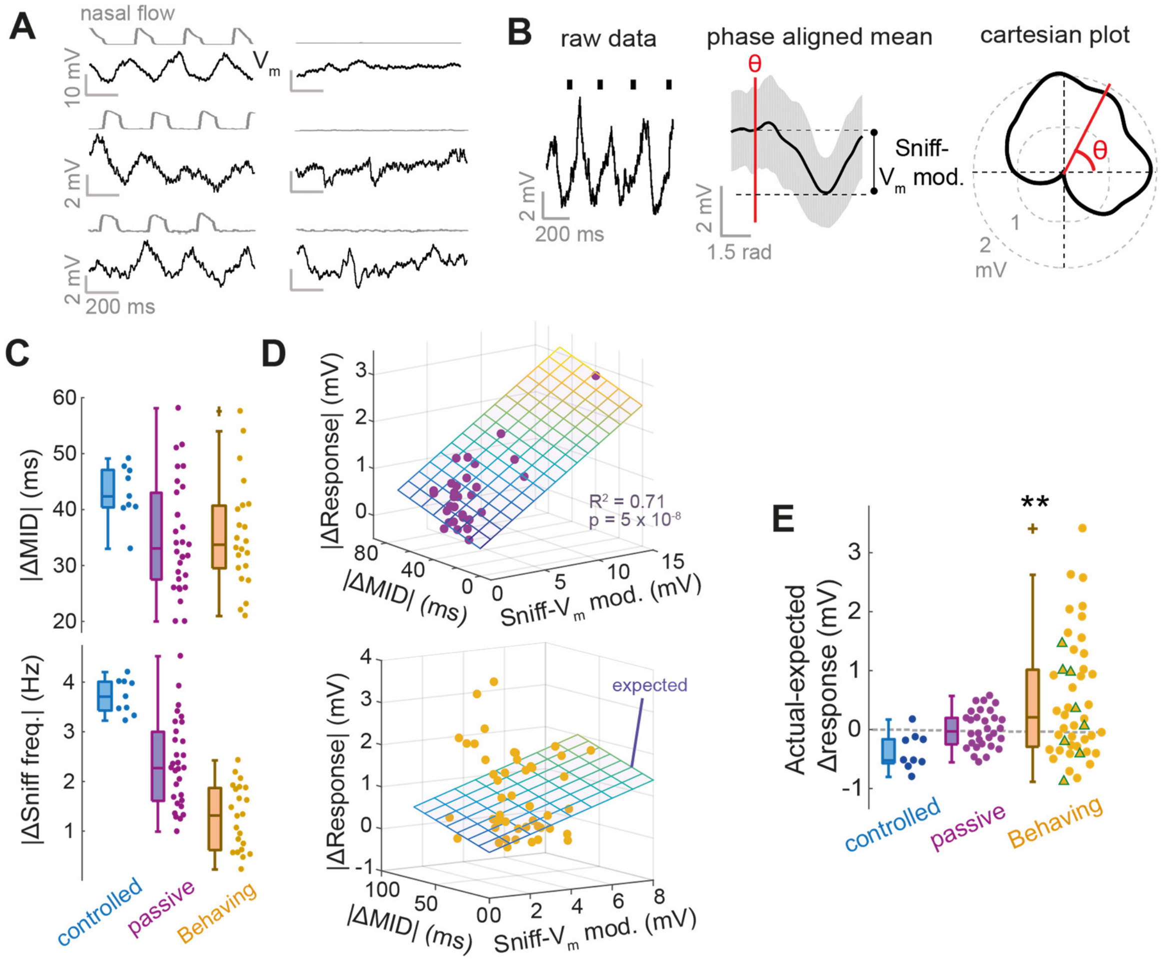

Figure S11. Additional data for behavioral state comparison of sniff changes, related to Figure 6.

(**A**) Example V_m traces from MTCs recorded in anaesthetized, tracheotomized mice during simulated rhythmic nasal flow (left) and no nasal flow (right) conditions. Note the lack of oscillations when flow is abolished. (**B**) Diagram to show calculation of phase preference and sniff-V_m modulation amplitude. Spike subtracted traces are considered only where sniffing is between 3 and 5 Hz (e.g. left plot showing V_m during 4 sniff cycles). Average phase-aligned V_m is then calculated (middle – shading shows SD). This waveform is then plotted in Cartesian coordinates (right), and the angle of the average vector, ϴ, is taken as the phase-preference of the cell. The sniff-V_m modulation amplitude is taken as the difference between the value of the phase-aligned V_m at this angle, and the minimal value (as indicated on middle plot). (**C**) Comparison of the magnitude of MID changes (top) and sniff frequency changes (bottom) in the three behavioral conditions, controlled-flow (anaesthetized), passive exposure (puff-evoked sniffing) and behaving mice (task engaged and learning mice, n = 26). (**D**) Above: scatterplot between the magnitude of MID change (|ΔMID|), sniff-V_m modulation amplitude, and the magnitude of response change (|Δresponse|) for all 30 cell-odor pairs recorded in passive mice when evoking sniff changes with puff stimuli. Plotted plane shows the multiple linear regression model explaining most variance in the data (|Δresponse| (in mV) = 0.088 + 0.16*T + 0.009*|ΔMID|, where T = sniff-V_m modulation amplitude in mV and |ΔMID| is in ms). Below: as for above, but for the behaving dataset (n = 46, 38 cell odor pairs from learning mice and 8 cell odor pairs from task engagement paradigm). Note the planar plot is not the multiple linear regression for this data (which revealed little correlation: R^2 = 0.09, p = 0.13), but for the passive data as above, and is used as a measure of 'expected' response change. (**E**) Plot comparing deviation of response change from the linear regression model (panel D), for anaesthetized (controlled-flow), passive and behaving mice. Behaving response changes are significantly larger than expected (p = 0.001, paired t-test). Controlled-flow response changes are significantly smaller than expected (p = 0.006, paired t-test).

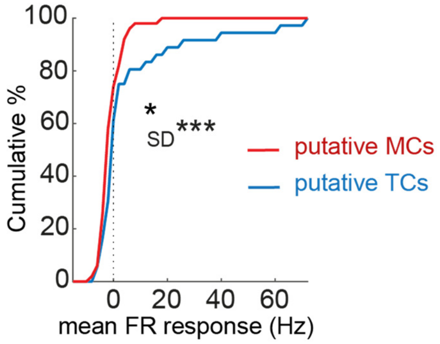

Figure S12. Comparison of average FR responses during odor stimuli for putative MCs and TCs, related to Figure 8.

Cumulative histogram to show average FR responses (averaged over full 2 s of the stimuli and all trials in the recording) for putative TCs (n = 36 cell-odor pairs) and putative MCs (n = 50 cell odor pairs), as defined by the sniff cycle phase preference, boundaries as shown in Figure 7F. pTC median FR response = -0.3Hz, IQR = -1.7 to 4.3Hz; pMC median response = -1.6Hz, IQR = -3.0 to 1.1Hz; $p = 6x10^{-17}$, Brown-Forsythe test; $p = 0.03$, Ranksum). Data comes from passive and learning mice.

9

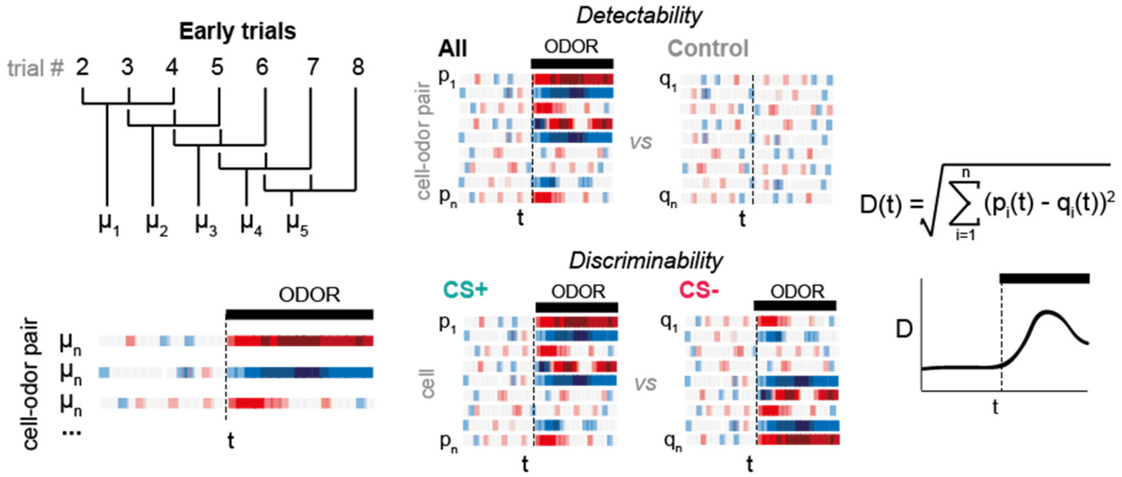

Figure S13. Diagram to show calculation of Euclidean distance values, detectability and discriminability, related to STAR methods.

For early and late trials from each cell-odor pair, 5 samples of 3 trials as shown (for early trials, they are drawn from trials 2 to 8, for mid-point trials, these are drawn from the mid-point ± 3 trials, and for late trials, these are drawn from the last 7 trials). Each sample of 3 is used to generate an average membrane potential (μ) waveform for each cell odor pair, depicted here in the form of a heatmap. One average for each cell-odor pair then forms a row in a population vector. Detectability is calculated as the Euclidean distance, D, at each time point, t, between the population vector containing odor responses, compared to a control population vector calculated in exactly the same way but using data from the inter-trial interval (no odor), triggered by a random inhalation. Discriminability is calculated from the Euclidean distance, D, at each time point, t, between population vectors for the CS+ and CS- independently. The process is repeated five times for each average, μ, to generate the five Euclidean distance waveforms.
